# Persuasive technologies design for mental and behavioral health platforms: A scoping literature review

**DOI:** 10.1371/journal.pdig.0000498

**Published:** 2024-05-16

**Authors:** Abdul Rahman Idrees, Robin Kraft, Agnes Mutter, Harald Baumeister, Manfred Reichert, Rüdiger Pryss

**Affiliations:** 1 Institute of Databases and Information Systems, Ulm University, Ulm, Germany; 2 Department of Clinical Psychology and Psychotherapy, Ulm University, Ulm, Germany; 3 Institute of Clinical Epidemiology and Biometry, University of Würzburg, Würzburg, Germany; 4 Institute of Medical Data Science, University Hospital Würzburg, Würzburg, Germany; Boston Consulting Group, KENYA

## Abstract

This review investigates persuasive design frameworks within eHealth, concentrating on methodologies, their prevalence in mental and behavioral health applications, and identifying current research gaps. An extensive search was conducted across 8 databases, focusing on English publications with full text available. The search prioritized primary research articles, post-2011 applications, and eHealth platforms emphasizing treatment or support. The inclusion process was iterative, involving multiple authors, and relied on detailed criteria to ensure the relevance and contemporaneity of selected works. The final review set comprised 161 articles, providing an overview of persuasive design frameworks in eHealth. The review highlights the state of the art in the domain, emphasizing the utilization and effectiveness of these frameworks in eHealth platforms. This review details the restricted adoption of persuasive design frameworks within the field of eHealth, particularly in the mental and behavioral sectors. Predominant gaps include the scarcity of comparative evaluations, the underrepresentation of tailored interventions, and the unclear influence of persuasive components on user experience. There is a notable requirement for further scrutiny and refinement of persuasive design frameworks. Addressing these concerns promises a more substantial foundation for persuasive design in eHealth, potentially enhancing user commitment and platform efficiency.

## Author summary

In our investigation, we examined a range of persuasive design frameworks that offer guidance on the development of persuasive systems. While exploring the literature, we analyzed the existing frameworks while also looking at how they are currently applied in health-related apps and platforms. We aimed to bridge the knowledge gap, giving system designers and developers a clearer picture of effective strategies in this space. We identified several areas with innovation potential, including some avenues where future work could enhance the effectiveness of digital health interventions. Our findings provide insights to those working in the eHealth industry and suggest that a deeper understanding and application of these persuasive design principles could lead to more impactful health outcomes. With this work, our goal is to inform the creation of eHealth tools that are more effective, thereby increasing their potential to enhance user health outcomes globally.

## Introduction

The term “eHealth” first appeared in the literature around 1999. Early users of the term defined eHealth as health services and/or health information delivered and accessed via the Internet. In 2000, [1] described eHealth specifically as “health care provided over the internet.” Over the past 2 decades, the field of information technology (IT) has progressed rapidly, and the concept of digital health has risen to greater prominence. Advances in fields such as artificial intelligence (AI), smartphones, wearables, and the Internet of Things (IoT) among others now offer more possibilities in the realm of healthcare delivery. [2] mentioned that digital health technologies are being used to assist in various health-related applications, including diagnosis, prevention, treatment, clinical decision support, care management, and care delivery. With the digital evolution, internet interventions have created more opportunities to deliver treatment to people with mental and behavioral health issues [3,4]. However, internet interventions often suffer from a lack of user engagement and high dropout rates. It has been observed that many users quit their interventions before finishing all their sessions [5]. While [6] highlighted adherence to a web-based self-help cognitive behavioral therapy (CBT) program, other research indicates different adherence patterns in guided eHealth interventions by mental health professionals [7]. A more detailed understanding of user behavior is needed, and the design of these platforms can significantly influence user adherence and engagement [8]. To address these challenges, [9] proposed persuasive design (PD) as an approach to influence user behavior in a way that motivates them to persist with their interventions. PD has its roots in Fogg’s Behavior Model (FBM), which postulates that behavior is influenced by 3 factors: motivation, ability, and triggers. Based on this model, PD is used to create systems that enhance user motivation, simplify abilities, and deliver timely triggers, aiming to guide users’ behavior or attitudes in a preferred direction without force or coercion [10]. This interdisciplinary field involves concepts of psychology, computer science, and design while incorporating principles from human–computer interaction and behavioral psychology to steer user actions. Techniques such as social proof (where users are influenced by the actions of others), commitment (where users are nudged to make small commitments that lead to larger actions), and gamification (use of game elements in non-game contexts) are often integrated within PD frameworks [11–13]. In eHealth applications, these techniques might appear as leaderboards comparing health behaviors, badges for health milestones, or reminders based on personal health goals. Within the eHealth field, PD can play a role in mitigating user dropout challenges by enhancing continuous engagement and adherence to online interventions [9]. This literature review aims to address the challenges in PD implementation within mental and behavioral eHealth platforms by mapping the existing knowledge. More specifically, it tries to answer the following questions:

1. What frameworks and methodologies are available for persuasive systems design?
2. How often is the use of frameworks and methodologies in eHealth apps addressed within the literature?
  2.1. What type of persuasive mental and behavioral eHealth apps are currently being studied in the literature and what persuasive features are they deploying?
3. What are the current research gaps in persuasive systems design for mental and behavioral eHealth platforms?

This review will analyze the body of literature, which discusses persuasive systems design frameworks and methodologies, with the aim of understanding their shortcomings and strengths. Hopefully, this will lead to more discussion and progress within the field. The subsequent sections are organized as follows: Section 2 outlines the methodology used in the review. The results of the review are presented in Section 3, while Section 4 discusses the results. Section 5 presents the limitation of this work, and Section 6 concludes the review.

## Methods

This section discusses the methodology used to review the literature. It starts with an overview and then discusses the procedure in detail.

### Overview

Several procedures were followed to ensure a high-quality review of the literature. First, the search included peer-reviewed journal papers, conferences, reports, and books. Eight databases were searched, including PubMed, IEEE Xplore digital library, Journal of Medical Internet Research, Google Scholar, ScienceDirect, ACM Digital Library, The Web of Science, and Oxford University Press. The literature search was conducted from February 2022 to December 2022.

Second, the reference section of each article was searched to identify more relevant articles. The following search terms were used: (‘persuasive design framework$’ OR ‘persuasive design strateg*’ OR ‘persuasive design architecture$’ OR ‘persuasive design methodolog*’) AND (‘eHealth platform$’ OR ‘eHealth app*’) AND (‘eHealth intervention$’ OR ‘internet intervention$’ OR ‘internet based intervention$’ OR ‘internet and mobile based intervention$’ OR ‘digital health intervention$’ OR ‘computerized CBT’) AND (‘persuasive design’ OR ‘persuasive technolog*’ OR ‘behaviour change’ OR ‘behavior change’ OR ‘gamification’). *$* represents zero or one character and * represents any number of characters including zero. For detailed reproducibility, the exact search string used is provided in S1 Text.

### Procedure

The search focused on sources published in English in which the full article was accessible. The first stage in the review was identifying relevant literature. The total number of identified articles reached 6,137. The next step in this stage was to remove any duplicates. The total number of removed duplicates was 1,161. The second stage was screening the articles by going over the titles, and, if the title was deemed relevant to the topic, the abstract would then be read. The purpose of this was to get an understanding of the contents of the paper and to run a first-phase relevance evaluation. This helped to decide if the paper should be included in the review. During this stage, 4,659 articles were excluded. The third stage involved going through the article itself to ensure it contained the full text. At this point, if it was not possible to obtain access to the full paper (behind a paywall/full text not available anymore), then it was dropped. Relevant papers found in the reference lists were also included. In the fourth stage, the documents were assessed based on the following eligibility criteria:

- Full text is in English: This ensured uniformity in comprehension and analysis.
- Unique articles: The primary goal was to pinpoint unique PD frameworks. Papers discussing the same framework without contributing new insights were omitted.
- Primary research papers: The aim was to center the review on original research and findings. Secondary research papers, which are syntheses of existing work, were excluded to maintain a focus on primary sources. This choice helps to capture firsthand insights and data rather than interpretations or aggregations.

In addition to the aforementioned criteria, when assembling the list of persuasive mental and behavioral IT solutions (defined as platforms or applications on mobile, web, or other platforms that offer interventions or self-help resources to assist those with mental or behavioral disorders, or aim to instigate behavioral changes in areas such as healthy lifestyles, sleep, or diet), the following exclusions were made:

- Articles that do not report the features of the IT solution delivering the intervention: A thorough understanding of the IT features included in the papers was required.
- Articles that only discuss designing a prototype without any implementation: The search focused on implementation rather than theoretical designs.
- Articles that use the same application that was used in other articles: To prevent redundancy, articles that examined applications previously detailed in other selected articles were excluded. This decision was made to maintain dataset integrity and offer new insights with each inclusion. The exclusion process was guided by the following set of applied criteria:
  - Comprehensiveness of application description: Preference was given to articles that provided detailed elaboration on the application’s design, its functionalities, and the underlying mechanics. Works offering a comprehensive account of the application’s design rationale and features were prioritized.
  - Clarification of persuasive strategies: Articles that offered clear classification, comprehensive explanation, and critical evaluation of persuasive strategies were prioritized.
  - Articles source and date: When confronted with multiple articles of comparable depth, the articles’ publisher became a determinant. Articles from reputable academic journals were favored. If this criterion, combined with the depth of detail, still posed a selection dilemma, the earlier article date became the decisive factor.
- Articles that describe applications focused only on auxiliary functions, like symptom detection, rather than direct treatment or support.
- Articles describing single-session applications: Single-session applications might not capture the full impact or advantages of PD.
- Articles that used applications dated before 2011: To remain contemporary, the review targeted articles involving applications from 2011 onwards. Earlier articles might be based on outdated technologies or paradigms, reducing their relevance to the current state of persuasive system design.

The initial screening of articles was undertaken by the first author, who evaluated titles, abstracts, and full-text articles for potential inclusion. After this, the coauthors reviewed the chosen articles, offering feedback. Their feedback was used to refine the inclusion criteria and finalize the list of articles included in this review.

Upon the selection of relevant articles, a structured data charting process was employed. The aim was to systematically extract and organize information from each article. The extracted details included core principles, strategies, and components of each PD framework, the year and geographical origin of the publication, framework mentions in other scholarly works, and specific categories or types of applications that employed the framework.

After data charting, the extracted information underwent a synthesis process to identify meaningful insights and patterns. The synthesis involved categorizing and analyzing the identified PD frameworks, providing historical and geographical context to each framework, assessing the practical implications based on mentions and applications in other works, and identifying gaps or areas underrepresented in the literature.

While the majority of articles presented conceptual frameworks or theoretical contributions, a subset of articles provided empirical evidence of the application of PD frameworks. These articles were subjected to a detailed critical appraisal. Specifically, these articles were evaluated on the following:

- Effectiveness evaluation: Classified outcomes as positive, negative, or mixed.
- Selection bias: Evaluated methods of participant selection.
- Reporting bias: Analyzed for overemphasis on positive outcomes.
- Detection bias: Assessed reliance on self-reports and tracking data.

**Fig 1:**
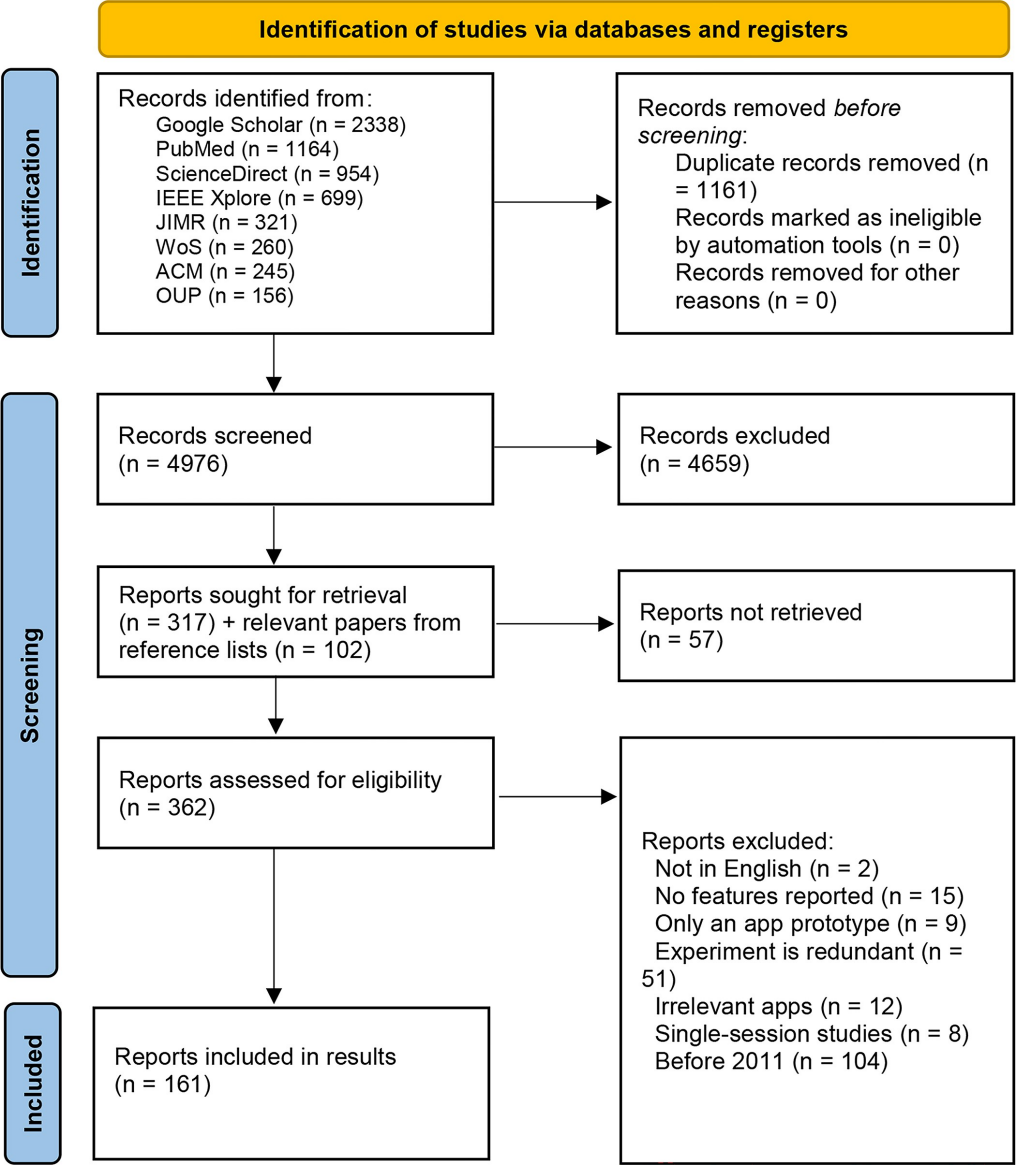
PRISMA flow diagram [14].

The review process led to the inclusion of 161 articles. This process is depicted in the PRISMA flowchart [14], as shown in Fig 1.

## Results

This section presents the main results of the literature review. First, it offers an overview of the frameworks and methodologies found in the literature, which are used to develop persuasive applications. It then compiles a list of the persuasive mental and behavioral health applications identified within the literature. Finally, it presents the research gaps identified in the review.

### Persuasive systems development

This subsection presents 15 frameworks and methodologies for the analysis, design, and development of persuasive systems. A brief description of each framework is available in Table 1.

**Table 1:** Summary of the PD frameworks.

Framework	Brief description
FBM [10]	Explores the interplay of motivation, ability, and triggers
PSD [13]	Enumerates 28 principles for behavior-influencing systems
Fogg’s 8-step [15]	Provides a structured process for goal definition and iterative design
Kimura and Nakajima [16]	Outlines strategies for conserving shared resources in collectivist settings
3D-RAB [17]	Prioritizes user attitude and behavior in design
CPP [18]	Emphasizes context-awareness in sustainable transport design
U-FADE [19]	Offers a 5-step design process addressing evolving user needs
Al-Ramahi et al. [20]	Grounded in user reviews, it highlights social and structural design elements
PEM [21]	Uses stages to chart user–behavior relationships and experience phases
Schneider et al. [22]	Uses the theory of planned behavior to study persuasive design motivation
Murillo-Munoz et al. [23]	Blends software development, user-centered design, and persuasive elements
Alpay et al. [24]	Merges CeHRes roadmap with FBM and PSD, emphasizing iterative eHealth design
Sari et al. [25]	Bridges PSD and BCW for mobile health applications
EMVE-DeCK [26]	A 7-step method harnessing social psychology for persuasive systems
Demonte and Souto [27]	Focuses on tailored mobile healthcare application design

CPP, Context-aware, Personalized, Persuasive; FBM, Fogg’s Behavioral Model; PD, persuasive design; PEM, Persuasive Experience Model; PSD, Persuasive System Design; U-FADE, Unified Framework for Analyzing, Designing, and Evaluating persuasive systems; 3D-RAB, 3-dimensional relationship between attitude and behavior.

In their work, Oinas-Kukkonen and Harjumaa [13] introduced the Persuasive System Design (PSD) model, providing a framework for the design and evaluation of persuasive systems. The PSD model is based on 7 foundational assumptions, which the authors categorically detail.

- User-Centric Assumptions: This category emphasizes the role of the end-user in the design and effectiveness of a persuasive system. The assumptions suggest that a system’s success relies on its ability to understand and adapt to the unique behaviors and perceptions of each user.
- Persuasion Strategy Assumptions: These assumptions stress the importance of carefully selecting and applying persuasion techniques. They also emphasize the importance of aligning strategies with the specific context and user profile in question.
- System Feature Assumptions: These assumptions are based on the fundamental features of a system and their role in influencing user behavior. They argue that depending on their design and implementation, these features can either facilitate or impede persuasive actions.

Additionally, Oinas-Kukkonen and Harjumaa [13] classifies system features into 4 comprehensive categories:

- Primary Task Features: These features concern the primary actions users undertake within the system. When incorporated correctly, users are effectively directed toward the desired behavioral outcomes.
- Dialogue Features: These features aim to optimize system–user interactions by providing feedback, reminders, and strategic reinforcements, supporting user engagement and adherence.
- System Credibility Features: These features help users view the system as authentic, reliable, and hence, persuasive.
- Social Support Features: These features utilize peer dynamics and collective collaboration

Each of these categories is supported by a set of design principles and guidelines that enable the model’s theoretical foundations to be effectively translated into practical applications.

In his research, Fogg presented the Behavior Model (FBM), a framework specifically designed to explain and affect behavior by combining 3 key factors: motivation, ability, and triggers [10]. Central to the FBM is the concept that for a behavior to change, an individual must concurrently possess the necessary motivation, the capability to perform the behavior, and a stimulus or trigger to initiate it. Should any of these elements be lacking or insufficient, the desired behavior is unlikely to materialize. In more details:

Motivation: Fogg [10] emphasizes the dynamic nature of motivation among individuals concerning certain behaviors. This motivation can be influenced by a range of factors, ranging from pleasure to pain, hope to fear, and even social like acceptance or rejection. Effective persuasive systems either opportunistically target individuals when their motivation peaks or try to amplify their existing motivation.
Ability: In Fogg’s paradigm, “ability” includes both tangible and intangible barriers like time, financial constraints, physical and cognitive efforts, and societal norms. Subsequently, persuasive systems should be designed to diminish these barriers, making tasks more accessible to the target demographic.
  - Triggers: These triggers, whether external, like notifications, or internal, such as specific emotional states or thinking patterns, incite the behavior. Their efficacy depends on their timely deployment, at precise moments when an individual is primed with both motivation and ability.

In his exploration of effective behavior change strategies, Fogg further developed his framework to include an 8-step design process aimed at guiding developers in the creation of persuasive systems [15]. These steps are detailed below:

1. Choose a simple behavior to target: The emphasis here is on granularity. By pinpointing a specific and manageable behavior, designers can effectively steer users without overwhelming or frustrating them.
2. Choose a receptive audience: Identifying an audience that is already interested in the desired behavior increases the likelihood of success.
3. Find what prevents the target behavior: By identifying and addressing the specific obstacles that hinder the desired behavior, designers can personalize interventions that directly counteract these obstacles.
4. Choose a familiar technology channel: Using platforms and mediums that the audience is already familiar with supports ease of use and reduces barriers to adoption.
5. Find relevant examples of persuasive technology: Studying established examples provides insights into effective strategies and minimizes uncertainty in the design process.
6. Imitate successful examples: Studying established strategies can speed up the design process and improve the acceptance rate among users.
7. Test and iterate quickly: Rapid testing cycles enable designers to obtain feedback, refine interventions, and adapt strategies quickly, enabling the system to stay agile and user-centric.
8. Expand on success: Once a strategy demonstrates success in a limited context, it can be scaled for broader impact and to validate the system’s effectiveness with a larger user base.

Kimura and Nakajima [16] presented a framework designed for collectivist societies. The framework is anchored in 5 strategic pillars:

1. Organizing groups: By creating groups around shared behavioral goals, the framework utilizes the power of collective motivation, influencing individuals towards desired behaviors.
2. Anonymity: In societies where reputation and societal perception hold significant weight, the framework acknowledges the importance of anonymity. This supports candid interactions, allowing users to transparently share their journey’s milestones and challenges.
3. Mutual surveillance: By making members’ actions transparent to peers, individuals are held accountable for their behavior and dedication to collective objectives.
4. Development of mutual aid: Kimura and Nakajima [16] emphasize the value of active collaboration, encouraging members to exchange insights, offer support, and collectively change their behavior.
5. Combine use of positive and negative feedback: This strategy offers a balanced feedback mechanism. It acknowledges achievements while also identifying areas for improvement.

Wiafe and colleagues [17] introduced the 3D-RAB model (3-dimensional relationship between attitude and behavior), which identifies a 3-dimensional relationship between the attitude towards the target behavior, the attitude towards changing/maintaining the current behavior, and the attitude towards the current behavior itself. The idea is to categorize the user’s state of cognitive dissonance based on the 3 elements of the 3D-RAB model. The following states of cognitive dissonance were identified:

1. Strong cognitive dissonance.
2. Moderate cognitive dissonance.
3. Weak cognitive dissonance.
4. No cognitive dissonance.

The Context-aware, Personalized, Persuasive (CPP) framework, as described by Prost and colleagues [18], presents a trilayered architecture optimized for the design and deployment of persuasive systems. This framework highlights the symbiotic relationship between targeted behaviors, user characteristics, and contextual variables, for a more comprehensive and adaptive approach to persuasion:

1. Target behavior layer: This layer determines the behaviors the system aims to promote or discourage.
2. User Layer: This layer is focused on customizing persuasive strategies according to individual user preferences, needs, and motivations.
3. Context-specific Layer: This layer refines the personalized strategies based on real-time contexts to ensure interventions remain relevant and effective.

Wiafe and colleagues [19] proposed the Unified Framework for Analyzing, Designing, and Evaluating persuasive systems (U-FADE), which draws upon the PSD model developed by Oinas-Kukkonen and Harjumaa [13]. The U-FADE framework comprises 5 steps:

1. Event Analysis: Allows designers to analyze the issues at hand.
2. Persuasive Strategy: Uses the feedback from the previous step to identify which factors may influence the target behavior.
3. Selection of System Features: Considers which persuasive strategies should be included from the PSD model.
4. Development and Implementation
5. Evaluating Change: Evaluates the behavior change resulting from the use of the system.

Al-Ramahi and colleagues [20] categorized the design of persuasive systems into 3 distinct domains: technical, structural, and social. Within these domains, they articulated several guiding principles:

1. Technical principles:
  - Self-Monitoring
  - Informative Presentation: Informative graphs for past activities
  - Effort Expectancy: Effort reduction
  - Persuasive Messages: Reminders
2. Structural Principles:
  - Connection with supporting elements in user’s context: data exchange between the app and supporting devices (if available)
3. Social Principles: Peer community support

Yu and Li [21] suggested a methodology called the Persuasive Experience Model (PEM) to aid in the design and implementation of persuasive systems. The process of creating a persuasive system—according to the PEM model—is divided into 6 stages: not known, recognized, interested, motivated, engaged, automated 1-obsessed, automated habitualized. According to Yu and Li [21], each one of the stages represents the psychological state of the targeted users of the system. System designers who are using the PEM model can map certain functions of their system into one of the stages of the model. However, mapping features and functions onto each stage of the PEM model is not a necessity. In addition, certain stages could be skipped based on the needs of the users and the system to be designed.

Schneider and colleagues [22] proposed a framework to assist with the design of persuasive applications. The framework consists of 2 principles that were derived from analyzing a popular fitness app. The principles are the following:

1. Perceived Behavioral Control: Convincing users of the ease of the task.
2. Support Flexibility: Consider the needs of different users who are classified into the following categories:
  - Followers: Those who enjoy seeing how other users are doing and benefit from mutual encouragement.
  - Hedonists: Those who do not like a competitive atmosphere and can lose interest quickly.
  - Achievers: Users who stay motivated and can sometimes be exposed to the risk of being too engaged.

Murillo-Munoz and colleagues [23] presented a framework combining 3 methodologies: Rapid Contextual Design (RCD), the PSD model, and Extreme Programming (XP). This structured framework presents a step-by-step process for the design of persuasive systems:

- Understanding key issues: An exploration of 7 concepts critical to the design of persuasive systems. These concepts provide a theoretical foundation for how technology influences user behavior.
- Contextual inquiry: A methodical data collection process followed by interpretation sessions to determine user profiles, goals, and challenges.
- Affinity diagramming: Organizing significant data points into coherent clusters for better understanding.
- Personas: Creating detailed user models to provide clearer direction for design.
- Analyze persuasion context: Determining intent, event, and strategy based on data collected.
- Wall walk and visioning: A participatory design phase, allowing for collaborative prototype creation.
- System qualities design: Employing the 4 major categories of PSD principles, with each principle further divided for nuanced application.
- Mockup evaluation: Testing paper prototypes in real-world scenarios for design validation.
- User stories selection: Transitioning to the XP phase, capturing design requirements into a narrative format.
- Story decomposition: Breaking narratives into specific tasks, for more efficient development.
- Release planning: Planning the development and deployment of individual tasks.
- Software cycle: A structured cycle of developing, integrating, and evaluating software releases.
- Software release: The culmination of the development process, preparing the system for real-world deployment.
- System evaluation: Assessing the system postdevelopment to ensure optimal performance.
- Outcome: Evaluating the resulting changes in user behavior and attitude postinteraction with the system.

Alpay and colleagues [24] utilized the PSD model created by Oinas-Kukkonen and Harjumaa [13] to propose a framework for the design of persuasive eHealth applications. This 3-part framework incorporates the CeHRes roadmap [25], the PSD model [13], and the FBM [10]. The framework requires the identification of stakeholders, which subsequently shapes the requirements definition. The design phase addresses user needs in terms of desired behavioral changes. The final phases—operationalization and evaluation—oversee the application’s rollout and subsequent performance assessment.

Sari and colleagues [26] used a combination of behavior change theories and persuasive technologies to propose a framework for the design of persuasive systems. The framework consists of 5 stages: define the behavioral problem, select the target behavior, evaluate target behavior and obstacles using Behavior Change Wheel (BCW) by Michie and colleagues [27], identify behavior change techniques, and, finally, identify the persuasive strategies from the PSD model by Oinas-Kukkonen and Harjumaa [13].

Oyibo [28] proposed the EMVE-DeCK framework, a tool designed to refine and customize persuasive technology. Based on the PSD model by Oinas-Kukkonen and Harjumaa [13], the proposed framework consists of 7 stages: explain, map, validate, explicate, design, change, and knowledge. The first 4 stages involve preparations, which include understanding the target behavior and mapping it onto the persuasive strategies of the PSD model, as well as attempting to understand the types of interface elements users wish to have. The last 3 stages focus on designing the system, deploying it, and documenting the process.

Demonte and Souto [29] introduced a design framework, created specifically for persuasive mobile health applications. Drawing inspiration from the PSD model [13] and FBM [10], the framework comprises 4 core components: Context Identification, Interface Design, Persuasion, and Suitability to Context. Each component is further divided into subcomponents to provide comprehensive guidance to system developers.

**Fig 2:**
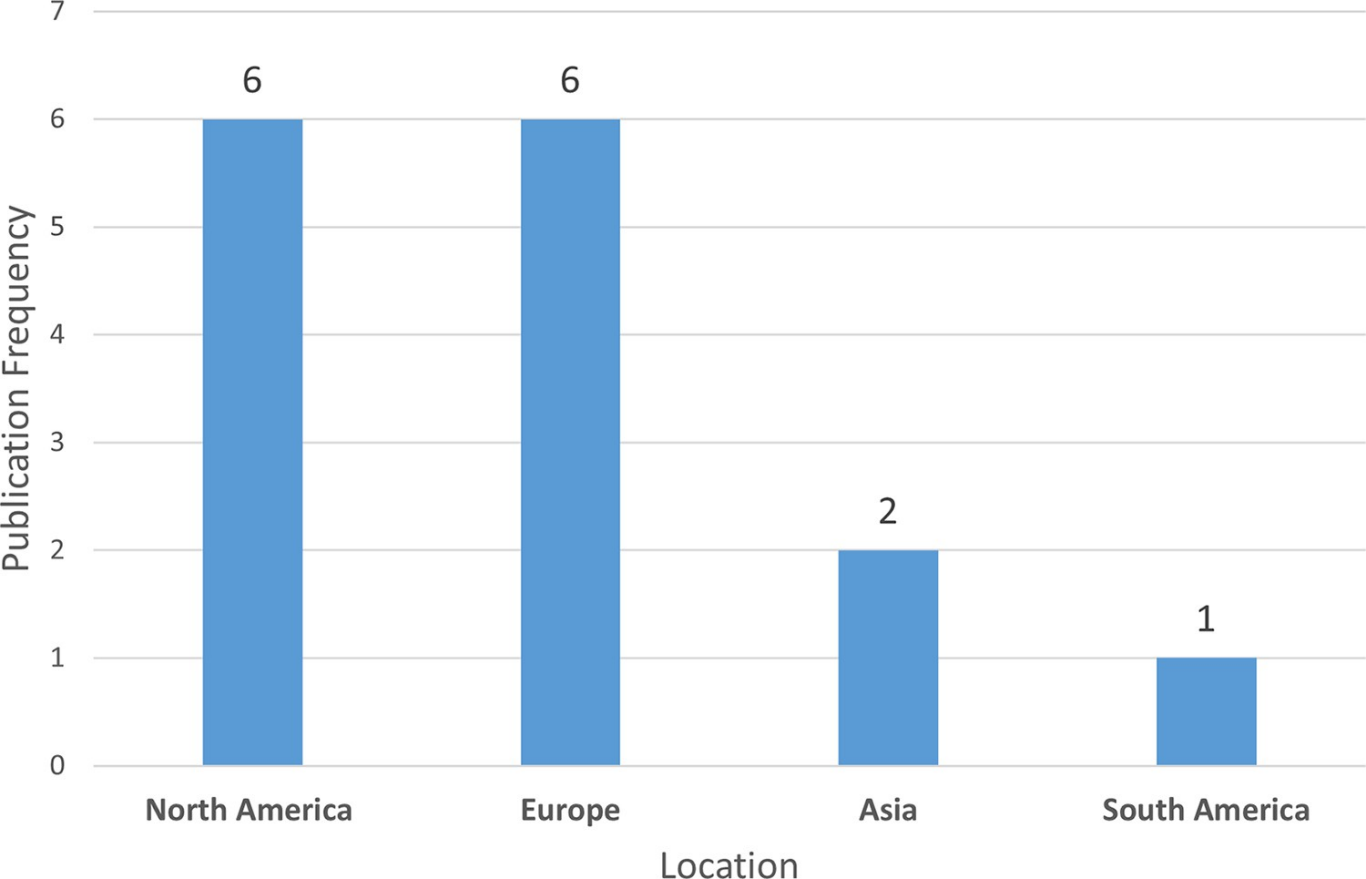
Geographical distribution of new PD framework creations by continent.

**Fig 3:**
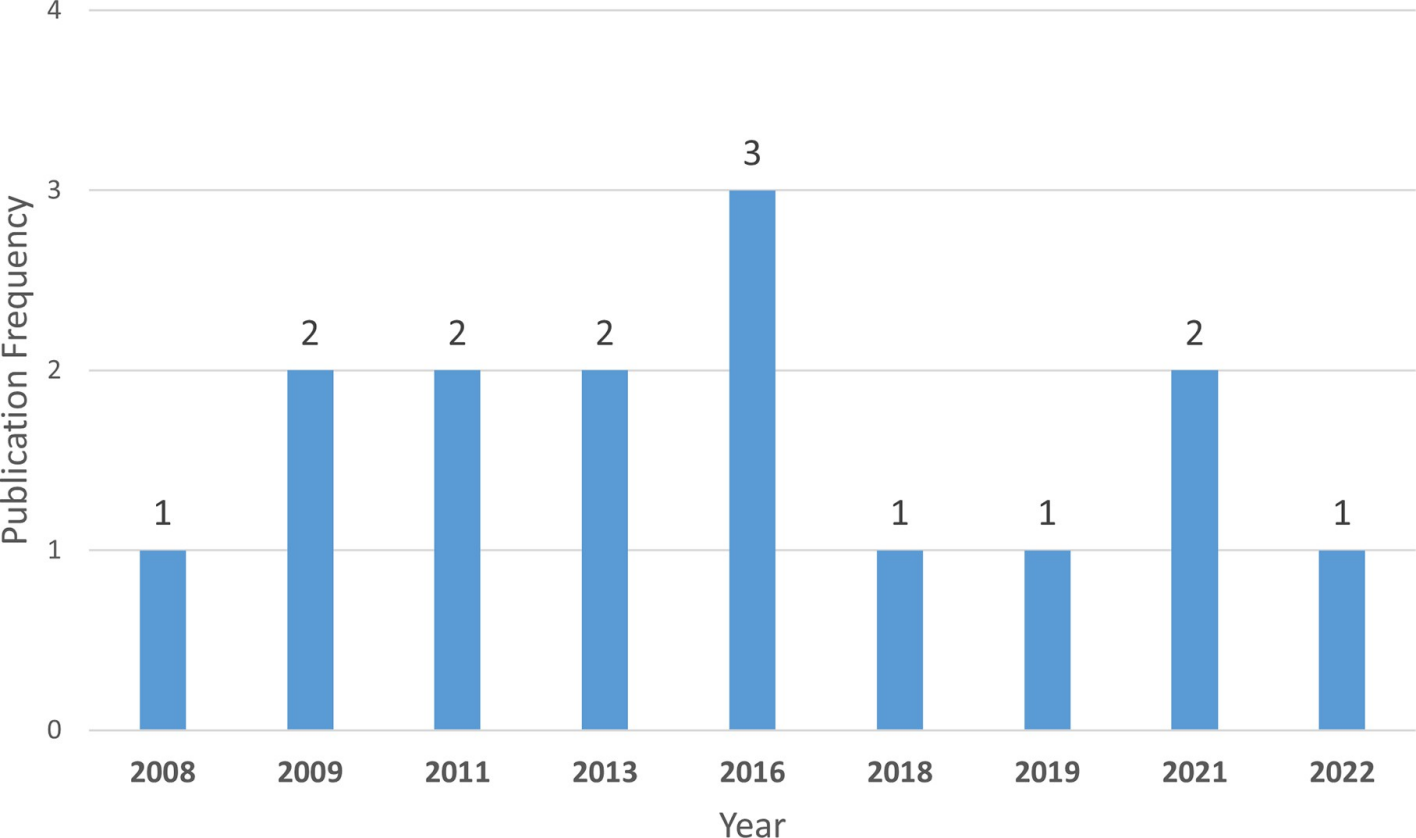
Yearly distribution of new PD framework publications.

Following the review of the above frameworks, Fig 2 displays trends in the creation of new PD frameworks across different geographical regions. Meanwhile, Fig 3 shows the yearly distribution of new PD framework publications. Additionally, Table 2 provides a comparative summary of the PD frameworks. These frameworks are evaluated based on the following criteria:

- The source of the framework’s publication: Journal, Conference, or Others.
- The framework’s grounding in a set of persuasive strategies.
- The framework’s consideration of the changing nature of user needs.
- The framework’s utilization of the PSD model.
- The framework’s domain.
- Whether the framework is derived from another source or is original.

**Table 2:** PD frameworks comparison.

Framework	Source	Strategies-based	Change of user’s needs	Utilize PSD	Domain	Derived vs. Original
FBM [10]	Others	-	-	-	Generic	Original
PSD [13]	Journal	✓	-	NA*	Generic	Original
Fogg’s 8-step [15]	Others	-	-	-	Generic	Derived
Kimura and Nakajima [16]	Journal	-	-	-	Collectivist cultures	Original
3D-RAB [17]	Conf.*	-	-	-	Generic	Original
CPP [18]	Conf.	✓	✓	-	Generic	Original
U-FADE [19]	Journal		-	✓	Generic	Derived
Al-Ramahi [20]	Conf.	✓	✓	-	Generic	Original
PEM [21]	Conf.	-	✓	-	Generic	Original
Schneide et al. [22]	Conf.	-	-	-	Fitness apps	Original
Murillo-Munoz et al. [23]	Conf.	-	✓	✓	Generic	Derived
Alpay et al. [24]	Jornal	-	-	✓	eHealth apps	Derived
Sari et al. [26]	Conf.	-	✓	✓	mHealth apps	Derived
EMVE-DeCK [28]	Conf.	✓	✓	✓	Generic	Derived
Demonte and Souto [29]	Journal	✓	✓	✓	mHealth apps	Original

*Conf., Conference; NA, Not applicable.

CPP, Context-aware, Personalized, Persuasive; FBM, Fogg’s Behavioral Model; PD, persuasive design; PEM, Persuasive Experience Model; PSD, Persuasive System Design; U-FADE, Unified Framework for Analyzing, Designing, and Evaluating persuasive systems; 3D-RAB, 3-dimensional relationship between attitude and behavior.

### Persuasive systems frameworks usage

One-hundred and forty-six (146) applications were identified in the literature between the years 2011 and 2022 that employed PD and behavior change techniques. The applications are grouped under different categories, as shown in Table 3.

**Table 3:** Apps categories.

Category	No. of Apps
Well-being	32
General behavior change	29
Substance abuse	19
Promote physical activity	15
Healthy diet	12
Depression support	12
Stress management	7
Anxiety support	5
Bipolar disorder	3
Suicide prevention	3
BPD support	2
OCD support	2
Aftercare	1
PTSD support	1
Dementia prevention	1
Health education	1
Obesity management	1

BPD, borderline personality disorder; OCD, obsessive-compulsive disorder; PTSD, posttraumatic stress disorder.

**Fig 4:**
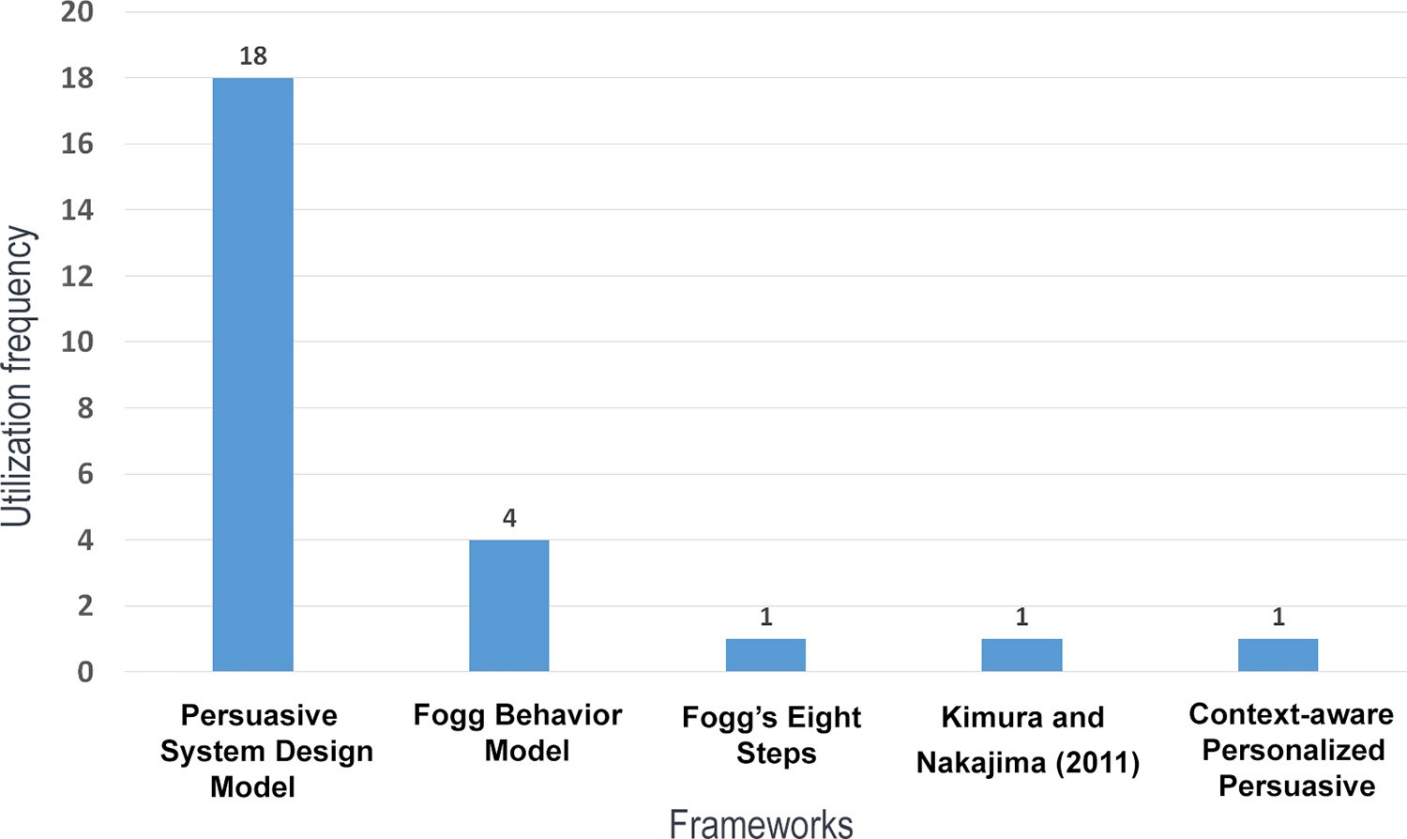
PD frameworks utilization’s frequencies.

PD frameworks were used by only 23 apps, with the PSD model by Oinas-Kukkonen and Harjumaa [13] being the most utilized—by 18 apps—as can be seen in Fig 4. The FBM was utilized 4 times, while Fogg’s 8-step methodology, the framework by Kimura and Nakajima [16], and the CPP framework by Prost and colleagues [18] were only used once. In some instances, a combination of frameworks was used to design an app. This was the case for Sankaran and colleagues [30], which used both the PSD model and FBM, and Karim and colleagues [31], which also utilized both frameworks during the design process.

A summarized representation of the frameworks and the corresponding publications is provided in Table 4 as well as in Table 5 along with their corresponding category. Furthermore, a list of papers found in the literature that utilize PD frameworks is presented in S1 Table.

**Table 4:** Summary of publications using PD frameworks.

Framework	Associated articles	No. of utilizations
PSD model	[30,31,34], [35], [32,36–45], [33,46,47]	18
FBM	[30,31,48,49]	4
Kimura and Nakajima (2011)	[16]	1
CPP	[50]	1
Fogg’s 8 steps	[51]	1

CPP, Context-aware, Personalized, Persuasive; FBM, Fogg’s Behavioral Model; PD, persuasive design; PSD, Persuasive System Design.

**Table 5:** Publications using PD frameworks.

Study	Framework	Category
[16]	Kimura and Nakajima	General behavior change
[30]	PSD model/FBM	General behavior change
[31]	PSD model/FBM	Substance abuse
[34]	PSD model	Promote physical activity
[50]	CPP	General behavior change
[51]	Fogg’s eight steps	Healthy eating
[35]	PSD model	General behavior change
[48]	FBM	Substance abuse
[32]	PSD model	Promote physical activity
[36]	PSD model	General behavior change
[37]	PSD model	Well-being
[38]	PSD model	General behavior change
[39]	PSD model	General behavior change
[40]	PSD model	General behavior change
[41]	PSD model	Well-being
[42]	PSD model	Bipolar disorder
[43]	PSD model	General behavior change
[44]	PSD model	Well-being
[45]	PSD model	Promote physical activity
[49]	FBM	General behavior change
[46]	PSD model	General behavior change
[47]	PSD model	General behavior change
[33]	PSD model	General behavior change

CPP, Context-aware, Personalized, Persuasive; FBM, Fogg’s Behavioral Model; PD, persuasive design; PSD, Persuasive System Design.

**Fig 5:**
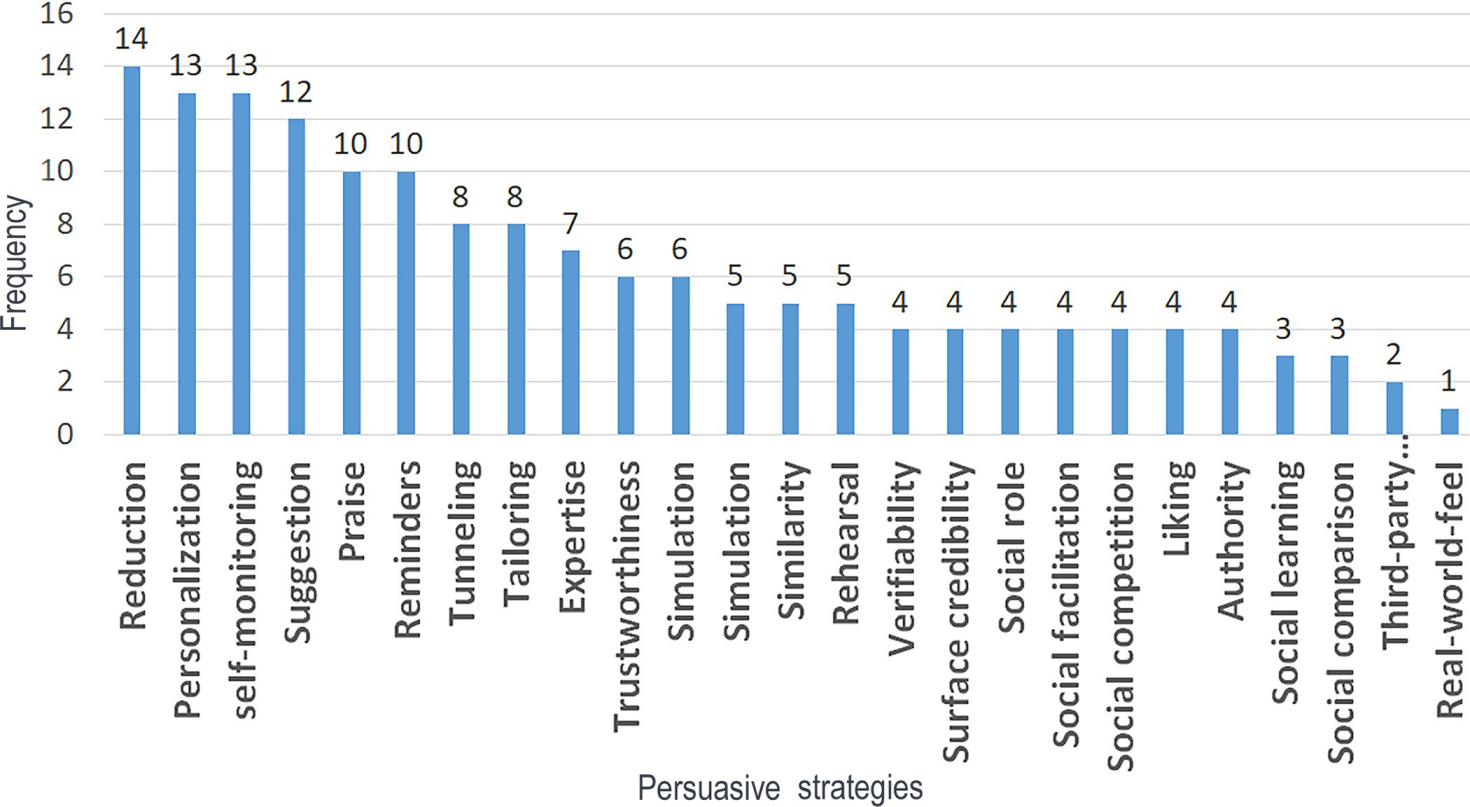
PSD persuasive strategies frequencies.

Among the apps that employed the PSD model, “reduction” was deployed by 14 apps, making it the most utilized persuasive strategy. After this, “self-monitoring” and personalization proved to be the second most utilized persuasive strategies—both were deployed by 13 apps. Fig 5 shows the utilization frequency of the strategies from the PSD model from Table 5. On average, 6.3 persuasive features were used in the publications utilizing the PSD model listed in Table 5, with 14 being the highest number of utilizations within 1 application [32], and 3 being the lowest [33].

### Characteristics of studies utilizing PD frameworks

From the publications listed in Table 5, 8 did not conduct evaluations for their applications, while one more described a study protocol. Of the subsequent 15 publications, the breakdown includes 9 observational studies, 4 descriptive studies, a randomized controlled trial (RCT), and a cross-sectional study, engaging a total of 1,043 participants.

While some publications failed to offer an exhaustive gender breakdown, available data enumerate 166 males and 836 females. Furthermore, participant age details fluctuated among studies: while some omitted age specifics, others employed age brackets. For context, average participant ages were as follows: 31.6 years in Fenicio and Calvary’s study [48], 55.5 years in that by Mohadis and colleagues [32], 45.5 years in Renfrew and colleagues’ study [44], and 29.84 years in the work of Jaffar and colleagues [33]. The evaluation of each study hinged on 2 primary criteria: the outcome—categorized as mixed, positive, or negative—and the inherent risk of bias. The biases considered include the following:

- Selection Bias: Arises from the methods employed for participant selection.
- Reporting Bias: Arises when studies predominantly emphasize positive outcomes and feedback
- Detection Bias: Arises due to the reliance on self-reported methods and application tracking data.

For detailed information, refer to S3 Table.

### Gaps in the literature

Based on the papers reviewed and the preceding results, several gaps have been identified. For a concise overview, refer to Table 6, which summarizes these gaps alongside associated publications. The identified gaps are presented below.

**Table 6:** Statistics of gaps identified in literature review.

Gap identified	Percentage	Publications	Gaps
Persuasive strategies comparison	0%	-	
Social support	3.7%	[16,32,37,51–53]	Families, couples, class room support
Usability vs. persuasiveness	1.2%	[53,54]	eHealth context
Tailored persuasive strategies	3.1%	[31,32,39,50,55]	Different backgrounds, users with disabilities
Employing AI and ML	3.1%	[56–60]	Personalized interactions
Negative effects of PD	0.6%	[34]	eHealth context

AI, artificial intelligence; ML, machine learning; PD, persuasive design.

**Comparing similar persuasive strategies against each other:** In the context of eHealth platforms, there is a noticeable gap in studies that conduct head-to-head comparisons of various persuasive strategies. This observation highlights a need for research that contrasts these strategies to understand their distinct and combined effects.

**Social support:** While certain mechanisms, notably “competition,” have been extensively studied, there is limited research exploring social support tailored for distinct cohorts, like couples or classmates.

**Understanding the influence of persuasion on applications usability:** The integration of persuasive strategies into eHealth platforms and its subsequent impact on usability has not been exhaustively studied. It is unclear whether the addition of persuasive strategies might compromise the user experience.

**Tailored persuasive strategies:** The literature suggests that many eHealth applications use broad persuasive strategies. However, there is a discernible gap in research focusing on strategies tailored to individual user preferences or demographics.

**Employing AI and machine learning (ML):** Future research should explore the potential of using AI and ML to personalize persuasive strategies in eHealth applications, including personalized lessons, suggestions, and reminders.

**Evaluating the negative effects of PD:** While much of the focus has been on the positive outcomes of PD, studies probing its potential adverse impacts, such as app usage addiction, are scarce.

## Discussion

This work has explored a variety of literature within the field of persuasive eHealth application development to assess the current progress of research in this area. This section first discusses persuasive systems frameworks, then moves on to discuss their usage in the literature, and, finally, elaborates on the research gaps identified in Section 3.

### Persuasive systems development evaluation analysis

It was noted that the PSD model proposed by Oinas-Kukkonen and Harjumaa [13] was generally present in other frameworks, as with Wiafe and colleagues [19], Murillo-Munoz and colleagues [23], and Alpay and colleagues [24]. This highlights the influential role of the PSD model in the development of persuasive systems. However, it can sometimes be noted that the PSD model’s persuasive strategies may be too similar or seem to overlap. Differentiating between constructs such as “rehearsal” and “simulation” poses inherent challenges, paralleled by the similarity observed between “social learning” and “cooperation,” both of which emphasize observational learning from peers.

Moreover, it is conceivable that deploying specific persuasive strategies might yield counterproductive outcomes in certain contexts. For instance, the concept of “tunneling” described by Fogg [11] involves directing users through a predetermined sequence of tasks. While this can optimize task efficiency and support a uniform user experience, it might also inadvertently constrain user autonomy. The limitations of “tunneling” become evident in the persuasive game “Smoke?” by Khaled and colleagues [61], where its application obscured feedback and status indicators, impeding optimal gameplay. This underscores the importance of carefully balancing “tunneling” with other persuasive strategies, such as conditioning, self-monitoring, and suggestion, to effectively maintain user attention and engagement.

The CPP framework proposed by Prost and colleagues [18] focuses on 2 elements: the user and the context of the application. Initially, designers should choose their persuasive strategies and then tailor these strategies to the user types expected to engage with the application and the context in which it will be used. The persuasive strategies suggested in the use case presented by Prost and colleagues [18] closely resemble those of the PSD model. However, the CPP framework differs from the PSD model in that it allows system designers more flexibility and does not confine them to a predefined set of persuasion strategies.

Building on this, the PSD model by Oinas-Kukkonen and Harjumaa offers a structured approach to persuasive design. One of its main components, the primary task support, includes principles such as “tailoring” and “personalization.” This mirrors the CPP’s recommendation to adapt strategies according to user and context. Furthermore, both frameworks highlight the significance of the user’s role in the persuasive process. Another similarity is the attention both frameworks give to the application’s environment or context. Just as the CPP advises designers to consider the application’s intended context, the PSD model’s dialogue assistance and system credibility principles emphasize the importance of creating a trustworthy and engaging environment for the user. In essence, while each framework presents its methodologies, the underlying theme is consistent: the emphasis on understanding the user, tailoring strategies, and considering the application’s context for more effective persuasion.

The U-FADE framework proposed by Wiafe and colleagues [19] incorporates the PSD model but also offers designers an iterative approach to refine the persuasive strategies employed in the system. However, Wiafe and colleagues applied the PSD model in its original form, suggesting that any limitations inherent to the PSD model could similarly impact the U-FADE framework. Like U-FADE, the frameworks proposed by Murillo-Munoz and colleagues [23] and Alpay and colleagues [24] also utilize the PSD model without proposing enhancements. Additionally, the design process proposed by Murillo-Munoz and colleagues is rather long and complex.

The EMVE-DeCK framework, as introduced by Oyibo [28], bears similarities to the framework introduced by Wiafe and colleagues [19]. Both methodologies begin with an analysis of the target behavior, followed by the selection of pertinent persuasive strategies from the PSD model and a subsequent evaluation of the applied implementation. However, a distinctive characteristic of Oyibo’s approach [28] lies in the inclusion of additional phases postimplementation, including deployment and comprehensive documentation.

The PEM model, presented by Yu and Li [21], deviates significantly from other extant frameworks. Unlike conventional models that regard the target behavior as a singular entity—evident in frameworks such as [17,19,23,28]—the PEM model aligns more with Fogg’s 8-step approach [15]. Here, the emphasis shifts to microbehavioral alterations, wherein the overarching target behavior is segmented into smaller, actionable objectives, facilitating nuanced analysis and targeted intervention.

In general, there is a clear similarity among many of the frameworks and methodologies found in the literature. Notably, 5 of these frameworks use the PSD model by Oinas-Kukkonen and Harjumaa [13] as a foundation to align persuasive strategies with the target behavior. Even for frameworks that do not adopt the PSD model directly, similarities are evident. This is the case with the works of Al-Ramahi and colleagues [20] and Demonte and Souto [29]. Both these publications emphasize the importance of understanding the context of a persuasive system, which is a key principle of the PSD model. The approach of Al-Ramahi and colleagues [20], based on user feedback and grounded theory, aligns well with the user-focused and context-sensitive aspects of the PSD model. Similarly, the framework by Demonte and Souto [29] combines context, interface design, and persuasive components, which matches the PSD model’s integrated approach to designing persuasive systems. The strong focus on context in the PSD model is also found in the framework created by Demonte and Souto [29].

It is also notable that both Fogg’s FBM [10] and his 8-step methodology [15] are oriented towards facilitating the user’s tasks. It can be argued that Fogg’s 8-step methodology is an evolved and more nuanced exposition of the FBM as described in [10]. It is important to note that the 8-step design process described in [15] primarily serves as a procedural guideline rather than an exhaustive systematic engineering methodology.

All things considered, a combination of frameworks could prove a better solution than using a single framework. For example, combining Fogg’s 8-step model with the PSD model could offer designers greater flexibility, allowing them to focus on small behavior targets rather than one major aim. Each small goal can be analyzed and the appropriate strategies of the PSD model can be mapped onto it. However, as mentioned above, the PSD model, despite its popularity, can still be improved by consolidating similar strategies into one and minimizing overlap in strategies. Within the findings of this review, several key themes emerged as prevalent across the reviewed PD frameworks. These themes include User Understanding, System Interaction, Persuasion Techniques, and Social and Trust Factors. A detailed synthesis of how each framework aligns with these themes can be found in S2 Table.

### Persuasive systems frameworks usage

From the reviewed papers, it was found that the proposed frameworks in references [15,17,20–24,26,28,29] have not been employed in the design or development of persuasive systems up to the conclusion of 2022.

The CPP framework, as proposed by Prost and colleagues [18], saw a singular application as referenced in Schrammel and colleagues’ study [50]. Similarly, the framework described by Kimura and Nakajima [16] was only applied within its introductory publication [16]. Several factors might account for this underutilization of these frameworks. Some of these, notably by Alpay and colleagues [24], Sari and colleagues [26], Demonte and Souto [29], and Oyibo [28], emerged between 2019 and 2021. Considering the brief duration since their introduction, it is reasonable to suggest that researchers and practitioners may not have had sufficient opportunity for extensive application. The absence of illustrative practical examples or case studies demonstrating the utility of these frameworks may lead to reluctance among designers and developers regarding their adoption. It is also notable that numerous publications did not articulate their rationale for favoring specific frameworks. However, the widespread use of the PSD model by Oinas-Kukkonen and Harjumaa [13] might be a contributing factor to its popularity over other frameworks.

Furthermore, the PSD model provides designers with a great degree of flexibility since it allows developers to map their persuasive goals into the numerous persuasive strategies provided by the framework. Subsequently, the PSD model can be used as a blueprint rather than a step-by-step design methodology. It is also difficult to ignore the rather complex nature of the frameworks—in particular [17,19,23,27]. Such complexity might be a contributing factor to the lack of utilization among persuasive apps. More research is needed to develop the frameworks mentioned above to increase their capacity for practical application outside the research community. It may even be beneficial if a new, simpler, and more practical design framework is developed.

### Gaps in the literature

Based on the reviewed literature, the following gaps were identified.

#### Comparing similar persuasive strategies against each other

Based on the results in Section 3, it was noted that persuasive strategies are rarely applied alone, as in the case of [30,31], to name a few. Although there are several studies that have investigated the experience of digital health app users in relation to persuasiveness—Foster and colleagues [62], for example—there is still a lack of understanding regarding the influence of each individual principle on the overall user experience. Hamari and colleagues [63] also indicated that in most cases, persuasive systems are evaluated as a whole, without examining their individual persuasive strategies to understand the influence of each. In particular, the comparison of different persuasive strategies within the same context has not yet attracted significant attention from researchers. Such a comparison could help researchers understand which persuasive strategies best fit which use cases, resulting in more effective designs. Furthermore, understanding the long-term effects of different persuasive features is important as it aids in creating more sustainable results. For example, Delmas and Kohli [64] reported that notifications in the context of their application were not able to maintain long-term motivation for users. More attention should be paid to understanding how effective each persuasive feature can be when employed over extended periods.

#### Social support

The framework proposed by Kimura and Nakajima [16] provides a starting point for anyone designing a persuasive system that facilitates collaboration between users. The same can also be true for the social support principle introduced in the PSD model by Oinas-Kukkonen and Harjumaa [13]. However, several areas in relation to social support remain to be fully explored. Notably, there appears to be a gap in research pertaining to persuasive strategies tailored specifically for couples undergoing concurrent interventions. Given the unique dynamics, reciprocal influences, and shared life challenges of these users, devising strategies for couples might necessitate considerations distinct from those applied to individuals. Further, another aspect of social support that warrants further research is the persuasive impact of familial backing for individuals engaged in interventions or preventative programs. Additionally, the area of peer support, particularly within educational institutions, presents another potential area for exploration. Many nuanced aspects of social support remain largely uncharted territories for eHealth application developers and intervention designers.

#### Understanding the influence of persuasion on application usability

Another area that has not attracted much attention from researchers is the relationship between usability and persuasiveness within eHealth platforms. Only a few studies were found that explored this topic. A study by Freeney [65] revealed that increased usability enhances persuasion, but more persuasion does not necessarily lead to increased usability. Additionally, Freeney [56] suggested that mobile app designers might face a trade-off between usability and persuasion in certain scenarios. Oyibo and Vassileva [54] also researched the relationship between usability and persuasiveness for a fitness app, recommending that designers should focus on creating visually appealing user interfaces that function well. However, their research only focused on a few persuasive principles in the context of the fitness app. More research is needed to understand the influence of persuasive principles on the usability of eHealth platforms and how user acceptance is affected.

#### Tailored persuasive strategies

The majority of the research on persuasive eHealth applications did not differentiate participants according to age group, type of mental health disorder, region of residence, educational background, financial status, and other differentiating factors—for example, as seen in [66,67]. However, usability studies generally paid more attention to how personal differences between users could affect usability perception [68–71]. Oyibo [72] drew a similar conclusion. More research could be performed in this area, perhaps providing app designers with more insight into how to tailor persuasive technologies to specific demographics. Moshe and colleagues [73] were able to effectively predict which individuals would be at high risk of early dropout from a digital intervention using a combination of intervention usage and baseline characteristic data. This model could inform the tailoring and personalization of persuasive strategies for specific individuals, encouraging prolonged use of interventions. Additionally, the literature shows a notable gap regarding users with disabilities; almost no research addresses the needs of this group. More research is needed to understand how persuasive features could be implemented and adjusted to support users with disabilities.

#### Employing AI and ML

Out of the total publications mentioned in S1 Table, only 5 employed AI in their applications to enhance user experience. AI’s primary use cases included generating suggestions and feedback [56,57], predicting the occurrence of specific behaviors and alerting users [58], and processing language during user communication [59,60]. While AI finds extensive applications in domains like anomaly detection, behavioral analysis, event detection, and sentiment analysis [74], its integration in persuasive systems remains limited. Notably, social media platforms employ AI to curate personalized advertisements and tailored content [75]. Similarly, AI could augment the persuasiveness of eHealth applications, offering personalized praise and progress feedback without the necessity of a human supervisor. However, this raises significant ethical concerns. Replacing human supervisors with AI might deprive users of authentic empathy crucial in fields like healthcare. Furthermore, unchecked AI biases could result in potentially harmful suggestions or feedback, and the inherent impersonality of AI could challenge user trust. Hence, while further research into this area is promising, it must be undertaken with ethical considerations in mind.

#### Evaluating the negative effects of PD

As for negative considerations, many of the PD strategies can be used to exploit users and encourage addictive use of technology. A report by Kidron and colleagues [76] published in 2018 warned about the possible negative effects of employing PD principles aimed at people, with specific concern for children. The report mentioned several examples of video games and social media platforms where designers exploited PD principles to encourage repetitive use, leading to habitual daily use. A common example of this is the reward principle found in many social media platforms in the form of “likes” and “shares.” Notifications and reminders that push users to check their devices frequently throughout the day can also have the same persuasive effect. Kidron and colleagues [76] outlined several negative consequences of this on children, such as increased anxiety, aggression, social media addiction, and sleep deprivation. The report recommended several steps to alleviate the potential negative consequences of PD and to protect children from electronic device addiction. These included keeping notifications switched off by default, providing “save” buttons to help children return to their task instead of being forced to finish it, and avoiding personal data collection. Despite the findings presented in Kidron and colleagues’ study [76], there is still a lack of research into the possible negative effects of persuasive technologies and how users of such technologies could be exploited. This is in agreement with the findings from Nyström and Stibe [77].

## Limitations

This review presents several limitations. Firstly, the focus was on programs or platforms mentioned in the literature, which may result in the omission of platforms not discussed in published works. Consequently, an understanding of the development methodologies of these unmentioned platforms might be limited. Secondly, the inclusion of only English-written papers could lead to the overlooking of significant contributions in other languages. Thirdly, despite consulting a diverse set of databases, pertinent studies in journals or conference proceedings not indexed in these databases might have been missed. Additionally, while the keyword search was comprehensive, some relevant articles might have been excluded due to varied terminology or specific phrasing. Finally, although theses were included in the review scope, other forms of grey literature, such as unpublished studies, were not systematically addressed, which might omit valuable insights.

## Conclusions

The literature review evaluated PD frameworks, and their application, and highlighted research gaps. While 15 distinct PD frameworks were identified, only a fraction of the 146 applications across health and behavioral domains integrated these frameworks. Notable research gaps include direct comparisons of persuasive strategies, the role of social support in PD, balancing usability with persuasiveness, and the implications of integrating AI and ML. Ethical concerns around PD also demand attention. Future research should focus on these gaps, compare PD frameworks in practical settings, refine existing methodologies, and continuously monitor the ethical ramifications as the domain progresses.

## Supporting information

S1 Table Persuasive eHealth apps. (DOCX)

S2 Table Synthesis for the PD frameworks. (DOCX)

S3 Table Studies evaluation. (DOCX)

S1 Text Detailed search strings. (DOCX)
