# Supplementary material for: Persuasive technologies design for mental and behavioral health platforms: A scoping literature review
Source: PLOS Digit Health. 2024 May 16;3(5):e0000498. doi: 10.1371/journal.pdig.0000498 (PMC11098517; doi:10.1371/journal.pdig.0000498)
Supplement: S1 Table — (DOCX) [file pdig.0000498.s001.docx]

**S1 Table.** Persuasive eHealth apps

| **Publication** | **Category** | **Publication** | **Category** |
| --- | --- | --- | --- |
| [30] | General behavior change | [31] | Substance abuse |
| [32] | Promote physical activity | [33] | General behavior change |
| [34] | Promote physical activity | [35] | general behavior change |
| [36] | General behavior change | [37] | Depression management |
| [38] | General behavior change | [39] | General behavior change |
| [41] | Well-being | [42] | BPD |
| [43] | General behavior change | [44] | Well-being |
| [45] | Promote physical activity | [46] | General behavior change |
| [47] | General behavior change | [48] | Substance abuse |
| [49] | General behavior change | [50] | General behavior change |
| [51] | Healthy eating | [52] | Promote physical activity |
| [53] | Depression support | [55] | Promote physical activity |
| [57] | Obesity prevention | [58] | Healthy eating |
| [59] | Depression support | [60] | Obesity prevention |
| [64] | General behavior change | [73] | Promote physical activity |
| [78] | Substance abuse | [79] | Well-being |
| [80] | Well-being | [81] | General behavior change |
| [82] | Stress management | [83] | Promote physical activity |
| [84] | Promote physical activity | [85] | Promote physical activity |
| [86] | Well-being | [87] | Well-being |
| [88] | Substance abuse | [89] | Stress management |
| [90] | Promote physical activity | [91] | Well-being |
| [92] | Substance abuse | [93] | Healthy eating |
| [94] | Bipolar disorder | [95] | Anxiety support |
| [96] | Promote physical activity | [97] | Stress management |
| [98] | Well-being | [99] | Substance abuse |
| [100] | Depression support | [101] | Anxiety management |
| [102] | Substance abuse | [103] | BPD |
| [104] | Anxiety management | [105] | Well-being |
| [106] | General behavior change | [107] | Depression management |
| [108] | BPD | [109] | Healthy eating |
| [110] | Depression support | [111] | Well-being |
| [112] | Depression support | [113] | Aftercare |
| [114] | Substance abuse | [115] | Stress management |
| [116] | General behavior change | [117] | Obesity prevention |
| [118] | Depression support | [119] | PTSD support |
| [120] | Depression support | [121] | Healthy eating |
| [122] | Insomnia management | [123] | Obesity prevention |
| [124] | Well-being | [125] | General behavior change |
| [126] | Well-being | [127] | Well-being |
| [128] | Well-being | [129] | Well-being |
| [130] | General behavior change | [131] | General behavior change |
| [132] | Well-being | [133] | Depression support |
| [134] | Healthy eating | [135] | Healthy eating |
| [136] | Stress management | [137] | Anxiety support |
| [138] | Anxiety support | [139] | Substance abuse |
| [140] | General behavior change | [141] | Healthy eating |
| [142] | General behavior change | [143] | Obesity management |
| [144] | Stress management | [145] | Substance abuse |
| [146] | Substance abuse | [147] | Depression support |
| [148] | Well-being | [149] | Suicide prevention |
| [150] | Depression support | [151] | Insomnia management |
| [152] | Addiction prevention | [153] | Substance abuse |
| [154] | Well-being | [155] | General behavior change |
| [156] | Suicide prevention | [157] | Well-being |
| [158] | Well-being | [159] | Healthy eating |
| [160] | General behavior change | [161] | Obesity prevention |
| [162] | Well-being | [163] | Well-being |
| [164] | Dementia prevention | [165] | General behavior change |
| [166] | Healthy eating | [167] | Well-being |
| [168] | Well-being | [169] | Well-being |
| [170] | Substance abuse | [171] | OCD |
| [172] | Depression support | [173] | Well-being |
| [174] | Stress management | [175] | Substance abuse |
| [176] | General behavior change | [177] | Well-being |
| [178] | Healthy eating | [179] | OCD |
| [180] | Substance abuse | [181] | Obesity prevention |
| [182] | Substance abuse | [183] | Depression support |
| [184] | Substance abuse | [185] | Suicide prevention |
| [186] | BPD | [187] | Substance abuse |
| [188] | Health education | [189] | Healthy eating |
| [190] | Well-being | [191] | Anxiety management |
| [192] | General behavior change |  | |

**References**

30. Sankaran S, Frederix I, Haesen M, Dendale P, Luyten K, Coninx K. A grounded approach for applying behavior change techniques in mobile cardiac tele-rehabilitation. Proceedings of the 9th ACM International Conference on PErvasive Technologies Related to Assistive Environments. 2016. pp. 1–8. doi:10.1007/978-3-642-69746-3

31. Karim NSA, AlHarbi A, AlKadhi B, AlOthaim N. Mobile Application on Smoking Cessation Based on Persuasive Design Theory. PACIS. 2017. p. 179.

32. Mohadis HM, Mohamad Ali N, Smeaton AF. Designing a persuasive physical activity application for older workers: understanding end-user perceptions. Behaviour & information technology. 2016;35: 1102–1114. doi:10.1080/0144929X.2016.1211737

33. Jaffar A, Mohd-Sidik S, Foo CN, Admodisastro N, Abdul Salam SN, Ismail ND. Improving pelvic floor muscle training adherence among pregnant women: Validation study. JMIR Hum Factors. 2022;9: e30989. doi:10.2196/30989

34. Purpura S, Schwanda V, Williams K, Stubler W, Sengers P. Fit4life: the design of a persuasive technology promoting healthy behavior and ideal weight. Proceedings of the SIGCHI conference on human factors in computing systems. 2011. pp. 423–432. doi:10.1145/1978942.1979003

35. Woldemicael BK. Designing a BCSS for mobile devices: an application to help the chronically tardy. 2015.

36. Tikka P, Woldemicael B, Oinas-Kukkonen H. Building an App for Behavior Change: Case RightOnTime. BCSS@ PERSUASIVE. 2016. pp. 3–14.

37. Yap MBH, Lawrence KA, Rapee RM, Cardamone-Breen MC, Green J, Jorm AF. Partners in parenting: a multi-level web-based approach to support parents in prevention and early intervention for adolescent depression and anxiety. JMIR Ment Health. 2017;4: e8492. doi:10.2196/mental.8492

38. Akker R op, Klaassen R, Bul K, Kato PM, van der Burg G-J, di Bitonto P. Let them play: Experiences in the wild with a gamification and coaching system for young diabetes patients. Proceedings of the 11th EAI international conference on pervasive computing technologies for healthcare. 2017. pp. 409–418. doi:10.1145/3154862.3154931

39. Senette C, Buzzi MC, Paratore MT, Trujillo A. Persuasive design of a mobile coaching app to encourage a healthy lifestyle during menopause. proceedings of the 17th International Conference on Mobile and Ubiquitous Multimedia. 2018. pp. 47–58. doi:10.1145/3282894.3282899

41. Ladwa S, Grønli T-M, Ghinea G. Towards Encouraging a Healthier Lifestyle and Increased Physical Activity–An App Incorporating Persuasive Design Principles. International Conference on Human-Computer Interaction. 2018. pp. 158–172.

42. Fletcher K, Foley F, Thomas N, Michalak E, Berk L, Berk M, et al. Web-based intervention to improve quality of life in late stage bipolar disorder (ORBIT): randomised controlled trial protocol. BMC Psychiatry. 2018;18: 1–13. doi:10.1186/s12888-018-1805-9

43. Häkkä M. Designing a Persuasive Mobile Application for Sharing Food Between Students and Restaurants. 2019.

44. Renfrew ME, Morton DP, Morton JK, Hinze JS, Beamish PJ, Przybylko G, et al. A web-and mobile app–based mental health promotion intervention comparing email, short message service, and videoconferencing support for a healthy cohort: randomized comparative study. J Med Internet Res. 2020;22: e15592. doi:10.2196/15592

45. Oyebode O, Ganesh A, Orji R. TreeCare: development and evaluation of a persuasive mobile game for promoting physical activity. 2021 IEEE Conference on Games (CoG). 2021. pp. 1–8. doi:10.1109/CoG52621.2021.9619035

46. Berry A, McClellan C, Wanless B, Walsh N, others. A Tailored App for the Self-management of Musculoskeletal Conditions: Evidencing a Logic Model of Behavior Change. JMIR Form Res. 2022;6: e32669. doi:10.2196/32669

47. Merz M, Hurm M. Goal-Setting for Knowledge Documentation using Persuasive Systems Design-Selection and Implementation of Design Principles. HICSS. 2022. pp. 1–10.

48. Fenicio A, Calvary G. Persuasion through an ambient device: proof of concept and early evaluation of cregrette, a smoking cessation system. European Conference on Ambient Intelligence. 2015. pp. 252–267.

49. Akmal M, Niwanputri GS. Spoonful: Mobile Application for Reducing Household Food Waste using Fogg Behavior Model (FBM). 2021 International Conference on Data and Software Engineering (ICoDSE). 2021. pp. 1–6. doi:10.1109/ICoDSE53690.2021.9648506

50. Schrammel J, Busch M, Tscheligi M. Peacox-Persuasive Advisor for CO2-Reducing Cross-Modal Trip Planning. PERSUASIVE (Adjunct Proceedings). 2013.

51. Pereira C V, Figueiredo G, Esteves MGP, de Souza JM. We4Fit: A game with a purpose for behavior change. Proceedings of the 2014 IEEE 18th International Conference on Computer Supported Cooperative Work in Design (CSCWD). 2014. pp. 83–88.

52. King AC, Hekler EB, Grieco LA, Winter SJ, Sheats JL, Buman MP, et al. Harnessing different motivational frames via mobile phones to promote daily physical activity and reduce sedentary behavior in aging adults. PLoS One. 2013;8: e62613. doi:10.1371/journal.pone.0062613

53. Vlaescu G, Alasjö A, Miloff A, Carlbring P, Andersson G. Features and functionality of the Iterapi platform for internet-based psychological treatment. Internet Interv. 2016;6: 107–114. doi:10.1016/j.invent.2016.09.006

55. Haque MS, Kangas M, Jämsä T. A persuasive mHealth behavioral change intervention for promoting physical activity in the workplace: feasibility randomized controlled trial. JMIR Form Res. 2020;4: e15083. doi:10.2196/15083

57. Sefa-Yeboah SM, Osei Annor K, Koomson VJ, Saalia FK, Steiner-Asiedu M, Mills GA. Development of a Mobile Application Platform for Self-Management of Obesity Using Artificial Intelligence Techniques. Int J Telemed Appl. 2021;2021. doi:10.1155/2021/6624057

58. Juarascio AS, Goldstein SP, Manasse SM, Forman EM, Butryn ML. Perceptions of the feasibility and acceptability of a smartphone application for the treatment of binge eating disorders: Qualitative feedback from a user population and clinicians. Int J Med Inform. 2015;84: 808–816. doi:10.1016/j.ijmedinf.2015.06.004

59. Fitzpatrick KK, Darcy A, Vierhile M. Delivering cognitive behavior therapy to young adults with symptoms of depression and anxiety using a fully automated conversational agent (Woebot): a randomized controlled trial. JMIR Ment Health. 2017;4: e7785. doi:10.2196/mental.7785

60. Rahmanti AR, Yang H-C, Bintoro BS, Nursetyo AA, Muhtar MS, Syed-Abdul S, et al. SlimMe, a Chatbot With Artificial Empathy for Personal Weight Management: System Design and Finding. Front Nutr. 2022;9. doi:10.3389/fnut.2022.870775

64. Delmas MA, Kohli A. Can apps make air pollution visible? Learning about health impacts through engagement with air quality information. Journal of business ethics. 2020;161: 279–302. doi:10.1007/s10551-019-04215-7

73. Moshe I, Terhorst Y, Paganini S, Schlicker S, Pulkki-Råback L, Baumeister H, et al. Predictors of Dropout in a Digital Intervention for the Prevention and Treatment of Depression in Patients With Chronic Back Pain: Secondary Analysis of Two Randomized Controlled Trials. J Med Internet Res. 2022;24: e38261. doi:10.2196/38261

78. Gustafson DH, Boyle MG, Shaw BR, Isham A, McTavish F, Richards S, et al. An e-health solution for people with alcohol problems. Alcohol Research & Health. 2011;33: 327.

79. Lane N, Mohammod M, Lin M, Yang X, Lu H, Ali S, et al. Bewell: A smartphone application to monitor, model and promote wellbeing. 5th international ICST conference on pervasive computing technologies for healthcare. 2012. doi:10.4108/icst.pervasivehealth.2011.246161

80. Lehto T, Oinas-Kukkonen H, Pätiälä T, Saarelma O. Virtual Health Check and Coaching: Insights from the Consumers and Implications for Persuasive Design. Exploring the Abyss of Inequalities: 4th International Conference on Well-Being in the Information Society, WIS 2012, Turku, Finland, August 22-24, 2012 Proceedings 4. 2012. pp. 29–40.

81. Langrial S, Oinas-Kukkonen H, Wang S. Design of a web-based information system for sleep deprivation–A trial study. Exploring the Abyss of Inequalities: 4th International Conference on Well-Being in the Information Society, WIS 2012, Turku, Finland, August 22-24, 2012 Proceedings 4. 2012. pp. 41–51.

82. Drozd F, Raeder S, Kraft P, Bjørkli CA, others. Multilevel growth curve analyses of treatment effects of a Web-based intervention for stress reduction: randomized controlled trial. J Med Internet Res. 2013;15: e2570. doi:10.2196/jmir.2570

83. Harries T, Eslambolchilar P, Stride C, Rettie R, Walton S. Walking in the wild–Using an always-on smartphone application to increase physical activity. IFIP conference on human-computer interaction. 2013. pp. 19–36.

84. Glynn LG, Hayes PS, Casey M, Glynn F, Alvarez-Iglesias A, Newell J, et al. SMART MOVE-a smartphone-based intervention to promote physical activity in primary care: study protocol for a randomized controlled trial. Trials. 2013;14: 1–7. doi:10.1186/1745-6215-14-157

85. Van Dantzig S, Geleijnse G, Van Halteren AT. Toward a persuasive mobile application to reduce sedentary behavior. Pers Ubiquitous Comput. 2013;17: 1237–1246. doi:10.1007/s00779-012-0588-0

86. Duncan M, Vandelanotte C, Kolt GS, Rosenkranz RR, Caperchione CM, George ES, et al. Effectiveness of a web-and mobile phone-based intervention to promote physical activity and healthy eating in middle-aged males: randomized controlled trial of the ManUp study. J Med Internet Res. 2014;16: e3107. doi:10.2196/jmir.3107

87. Heffernan KJ, Chang S, Maclean ST, Callegari ET, Garl SM, Reavley N, et al. The potential of eHealth apps to support targeted complex health messages. Journal of General Practice. 2014. doi:10.4172/2329-9126.1000182

88. Bricker JB, Mull KE, Kientz JA, Vilardaga R, Mercer LD, Akioka KJ, et al. Randomized, controlled pilot trial of a smartphone app for smoking cessation using acceptance and commitment therapy. Drug Alcohol Depend. 2014;143: 87–94. doi:10.1016/j.drugalcdep.2014.07.006

89. Ly KH, Asplund K, Andersson G. Stress management for middle managers via an acceptance and commitment-based smartphone application: A randomized controlled trial. Internet Interv. 2014;1: 95–101. doi:10.1016/j.invent.2014.06.003

90. Thorsteinsen K, Vittersø J, Svendsen GB. Increasing physical activity efficiently: an experimental pilot study of a website and mobile phone intervention. Int J Telemed Appl. 2014;2014. doi:10.1155/2014/746232

91. Kang H, Park HA. Development of hypertension management mobile application based on clinical practice guidelines. Digital Healthcare Empowering Europeans. IOS Press; 2015. pp. 602–606. doi:10.3233/978-1-61499-512-8-602

92. Bertholet N, Cunningham JA, Faouzi M, Gaume J, Gmel G, Burnand B, et al. Internet-based brief intervention for young men with unhealthy alcohol use: A randomized controlled trial in a general population sample. Addiction. 2015;110: 1735–1743. doi:10.1111/add.13051

93. Tregarthen JP, Lock J, Darcy AM. Development of a smartphone application for eating disorder self-monitoring. International Journal of Eating Disorders. 2015;48: 972–982. doi:10.1002/eat.22386

94. Hidalgo-Mazzei D, Mateu A, Reinares M, Undurraga J, Bonnin C del M, Sánchez-Moreno J, et al. Self-monitoring and psychoeducation in bipolar patients with a smart-phone application (SIMPLe) project: design, development and studies protocols. BMC Psychiatry. 2015;15: 1–9. doi:10.1186/s12888-015-0437-6

95. Miloff A, Marklund A, Carlbring P. The challenger app for social anxiety disorder: New advances in mobile psychological treatment. Internet Interv. 2015;2: 382–391. doi:10.1016/j.invent.2015.08.001

96. Walsh JC, Corbett T, Hogan M, Duggan J, McNamara A. An mHealth intervention using a smartphone app to increase walking behavior in young adults: a pilot study. JMIR Mhealth Uhealth. 2016;4: e5227.

97. Heber E, Lehr D, Ebert DD, Berking M, Riper H, others. Web-based and mobile stress management intervention for employees: a randomized controlled trial. J Med Internet Res. 2016;18: e5112.

98. Schuurmans J, van der Linden JL, van Ballegooijen W, Ruwaard J, Stek ML, Smit JH, et al. Tablet-based support for older adults with severe mood disorders treated in an ambulatory geriatric psychiatry setting: Protocol of a feasibility study of the eCare@ Home platform. Internet Interv. 2016;6: 22–28. doi:10.1016/j.invent.2016.09.001

99. Arnaud N, Baldus C, Elgán TH, De Paepe N, Tønnesen H, Csémy L, et al. Effectiveness of a web-based screening and fully automated brief motivational intervention for adolescent substance use: a randomized controlled trial. J Med Internet Res. 2016;18: e4643. doi:10.2196/jmir.4643

100. Frazier P, Richards D, Mooney J, Hofmann SG, Beidel D, Palmieri PA, et al. Acceptability and proof of concept of internet-delivered treatment for depression, anxiety, and stress in university students: protocol for an open feasibility trial. Pilot Feasibility Stud. 2016;2: 1–9. doi:10.1186/s40814-016-0068-9

101. Vigerland S, Ljótsson B, Thulin U, Öst L-G, Andersson G, Serlachius E. Internet-delivered cognitive behavioural therapy for children with anxiety disorders: A randomised controlled trial. Behaviour research and therapy. 2016;76: 47–56.

102. Businelle MS, Ma P, Kendzor DE, Frank SG, Vidrine DJ, Wetter DW. An ecological momentary intervention for smoking cessation: evaluation of feasibility and effectiveness. J Med Internet Res. 2016;18: e6058. doi:10.2196/jmir.6058

103. Rizvi SL, Hughes CD, Thomas MC. The DBT Coach mobile application as an adjunct to treatment for suicidal and self-injuring individuals with borderline personality disorder: A preliminary evaluation and challenges to client utilization. Psychol Serv. 2016;13: 380. doi:10.1037/ser0000100

104. Whiteside SPH. Mobile device-based applications for childhood anxiety disorders. J Child Adolesc Psychopharmacol. 2016;26: 246–251. doi:10.1089/cap.2015.0010

105. Krishnamurti T, Davis AL, Wong-Parodi G, Fischhoff B, Sadovsky Y, Simhan HN, et al. Development and testing of the Myhealthypregnancy app: a behavioral decision research-based tool for assessing and communicating pregnancy risk. JMIR Mhealth Uhealth. 2017;5: e7036. doi:10.2196/mhealth.7036

106. Yang HJ, Kang J-H, Kim OH, Choi M, Oh M, Nam J, et al. Interventions for preventing childhood obesity with smartphones and wearable device: a protocol for a non-randomized controlled trial. Int J Environ Res Public Health. 2017;14: 184. doi:10.3390/ijerph14020184

107. Addepally SA, Purkayastha S. Mobile-application based cognitive behavior therapy (CBT) for identifying and managing depression and anxiety. International Conference on Digital Human Modeling and Applications in Health, Safety, Ergonomics and Risk Management. 2017. pp. 3–12.

108. Suñol J, Panisello JM, Castell E, Tárraga JP, Sánchez C, Pérez V. Medtep DBT”: A dialectical behavior therapy native app and web platform for borderline personality disorder patients and their therapists. Univers J Public Health. 2017;5: 110–118. doi:10.13189/ujph.2017.050305

109. Freyne J, Yin J, Brindal E, Hendrie GA, Berkovsky S, Noakes M. Push notifications in diet apps: influencing engagement times and tasks. Int J Hum Comput Interact. 2017;33: 833–845. doi:10447318.2017.1289725

110. Schlosser DA, Campellone TR, Truong B, Anguera JA, Vergani S, Vinogradov S, et al. The feasibility, acceptability, and outcomes of PRIME-D: A novel mobile intervention treatment for depression. Depress Anxiety. 2017;34: 546–554. doi:10.1002/da.22624

111. Zhu J, Ebert L, Liu X, Chan SW-C. A mobile application of breast cancer e-support program versus routine care in the treatment of Chinese women with breast cancer undergoing chemotherapy: study protocol for a randomized controlled trial. BMC Cancer. 2017;17: 1–9. doi:10.1186/s12885-017-3276-7

112. Mohr DC, Tomasino KN, Lattie EG, Palac HL, Kwasny MJ, Weingardt K, et al. IntelliCare: an eclectic, skills-based app suite for the treatment of depression and anxiety. J Med Internet Res. 2017;19: e6645. doi:10.2196/jmir.6645

113. Willems RA, Mesters I, Lechner L, Kanera IM, Bolman CAW. Long-term effectiveness and moderators of a web-based tailored intervention for cancer survivors on social and emotional functioning, depression, and fatigue: randomized controlled trial. Journal of Cancer Survivorship. 2017;11: 691–703. doi:10.1007/s11764-017-0625-0

114. Brendryen H, Johansen A, Duckert F, Nesvåg S. A pilot randomized controlled trial of an internet-based alcohol intervention in a workplace setting. Int J Behav Med. 2017;24: 768–777. doi:10.1007/s12529-017-9665-0

115. Eklund C, Elfström ML, Eriksson Y, Söderlund A. Development of the web application My Stress Control—Integrating theories and existing evidence. Cogent Psychol. 2018;5: 1489457. doi:23311908.2018.1489457

116. Beleigoli AM, de Andrade A, Haueisen Diniz M de F, Alvares RS, Ribeiro AL. Online platform for healthy weight loss in adults with overweight and obesity-the “POEmaS” project: a randomized controlled trial. BMC Public Health. 2018;18: 1–7. doi:10.1186/s12889-018-5882-y

117. Huang C-Y, Yang M-C, Huang C-Y, Chen Y-J, Wu M-L, Chen K-W. A chatbot-supported smart wireless interactive healthcare system for weight control and health promotion. 2018 IEEE international conference on industrial engineering and engineering management (IEEM). 2018. pp. 1791–1795. doi:10.1109/IEEM.2018.8607399

118. Mehrotra S, Sudhir P, Rao G, Thirthalli J, Srikanth TK. Development and pilot testing of an internet-based self-help intervention for depression for Indian users. Behavioral Sciences. 2018;8: 36. doi:10.3390/bs8040036

119. Bauer AM, Hodsdon S, Bechtel JM, Fortney JC. Applying the principles for digital development: case study of a smartphone app to support collaborative care for rural patients with posttraumatic stress disorder or bipolar disorder. J Med Internet Res. 2018;20: e10048. doi:10.2196/10048

120. Carswell K, Harper-Shehadeh M, Watts S, van’t Hof E, Abi Ramia J, Heim E, et al. Step-by-Step: a new WHO digital mental health intervention for depression. Mhealth. 2018;4. doi:10.21037/mhealth.2018.08.01

121. Lau Y, Cheng LJ, Chi C, Tsai C, Ong KW, Ho-Lim SST, et al. Development of a healthy lifestyle mobile app for overweight pregnant women: qualitative study. JMIR Mhealth Uhealth. 2018;6: e9718. doi:10.2196/mhealth.9718

122. Pulantara IW, Parmanto B, Germain A. Development of a just-in-time adaptive mHealth intervention for insomnia: usability study. JMIR Hum Factors. 2018;5: e8905. doi:10.2196/humanfactors.8905

123. Carvalho M, Alves R, Reis C, Martinho R, Sousa P, Gaspar P. Teenpower: an integrated architecture for an mHealth platform designed for e-Empowering teenagers to prevent obesity: A showcase of the TeenPower platform. 2018 IEEE 20th International Conference on e-Health Networking, Applications and Services (Healthcom). 2018. pp. 1–4. doi:10.1109/HealthCom.2018.8531163

124. Lee RA, Jung ME. Evaluation of an mhealth app (destressify) on university students’ mental health: pilot trial. JMIR Ment Health. 2018;5: e8324. doi:10.2196/mental.8324

125. Murawski B, Plotnikoff RC, Rayward AT, Vandelanotte C, Brown WJ, Duncan MJ. Randomised controlled trial using a theory-based m-health intervention to improve physical activity and sleep health in adults: the Synergy Study protocol. BMJ Open. 2018;8: e018997. doi:10.1136/bmjopen-2017-018997

126. Pryss R, Reichert M, John D, Frank J, Schlee W, Probst T. A personalized sensor support tool for the training of mindful walking. 2018 IEEE 15th international conference on wearable and implantable Body Sensor Networks (BSN). 2018. pp. 114–117. doi:10.1109/BSN.2018.8329672

127. Kelders SM, Sommers-Spijkerman M, Goldberg J. Investigating the direct impact of a gamified versus nongamified well-being intervention: an exploratory experiment. J Med Internet Res. 2018;20: e9923. doi:10.2196/jmir.5112

128. Fanning J, Brooks AK, Ip E, Nicklas BJ, Rejeski WJ, others. A mobile health intervention to reduce pain and improve health (MORPH) in older adults with obesity: protocol for the MORPH trial. JMIR Res Protoc. 2018;7: e9712. doi:10.2196/resprot.9712

129. Bakker D, Kazantzis N, Rickwood D, Rickard N. Development and pilot evaluation of smartphone-delivered cognitive behavior therapy strategies for mood-and anxiety-related problems: MoodMission. Cogn Behav Pract. 2018;25: 496–514. doi:10.1016/j.cbpra.2018.07.002

130. Roncero M, Belloch A, Doron G, others. Can brief, daily training using a mobile app help change maladaptive beliefs? Crossover randomized controlled trial. JMIR Mhealth Uhealth. 2019;7: e11443. doi:10.2196/11443

131. Gallagher R, Chow C, Parker H, Neubeck L, Celermajer D, Redfern J, et al. Design and rationale of the MyHeartMate study: a randomised controlled trial of a game-based app to promote behaviour change in patients with cardiovascular disease. BMJ Open. 2019;9: e024269. doi:10.1136/bmjopen-2018-024269

132. Coelhoso CC, Tobo PR, Lacerda SS, Lima AH, Barrichello CRC, Amaro Jr E, et al. A new mental health mobile app for well-being and stress reduction in working women: randomized controlled trial. J Med Internet Res. 2019;21: e14269. doi:10.2196/14269

133. Goldin PR, Lindholm R, Ranta K, Hilgert O, Helteenvuori T, Raevuori A. Feasibility of a therapist-supported, mobile phone–delivered online intervention for depression: longitudinal observational study. JMIR Form Res. 2019;3: e11509. doi:10.2196/11509

134. Trojaniello D, Sacchitelli F, Massa E, Vismara P, Sanna A. A NOVEL WEB APPLICATION (FOODCOACH) TO MONITOR AND IMPROVE EATING BEHAVIORS: PILOT RANDOMIZED CONTROLLED TRIAL.

135. Rachakonda L, Kothari A, Mohanty SP, Kougianos E, Ganapathiraju M. Stress-Log: An IoT-based smart system to monitor stress-eating. 2019 IEEE International Conference on Consumer Electronics (ICCE). 2019. pp. 1–6. doi:10.1109/ICCE.2019.8661959

136. Huberty J, Green J, Glissmann C, Larkey L, Puzia M, Lee C, et al. Efficacy of the mindfulness meditation mobile app “calm” to reduce stress among college students: Randomized controlled trial. JMIR Mhealth Uhealth. 2019;7: e14273. doi:10.2196/14273

137. Heckendorf H, Lehr D, Ebert DD, Freund H. Efficacy of an internet and app-based gratitude intervention in reducing repetitive negative thinking and mechanisms of change in the intervention’s effect on anxiety and depression: results from a randomized controlled trial. Behaviour research and therapy. 2019;119: 103415. doi:10.1016/j.brat.2019.103415

138. Greer JA, Jacobs J, Pensak N, MacDonald JJ, Fuh C-X, Perez GK, et al. Randomized trial of a tailored cognitive-behavioral therapy mobile application for anxiety in patients with incurable cancer. Oncologist. 2019;24: 1111–1120. doi:10.1634/theoncologist.2018-0536

139. Bertholet N, Godinho A, Cunningham JA. Smartphone application for unhealthy alcohol use: pilot randomized controlled trial in the general population. Drug Alcohol Depend. 2019;195: 101–105.

140. Mortenson W Ben, Singh G, MacGillivray M, Sadeghi M, Mills P, Adams J, et al. Development of a self-management app for people with spinal cord injury. J Med Syst. 2019;43: 1–12. doi:10.1007/s10916-019-1273-x

141. Nour M, Chen J, Allman-Farinelli M, others. Young adults’ engagement with a self-monitoring app for vegetable intake and the impact of social media and gamification: feasibility study. JMIR Form Res. 2019;3: e13324. doi:10.2196/13324

142. Agarwal P, Mukerji G, Desveaux L, Ivers NM, Bhattacharyya O, Hensel JM, et al. Mobile app for improved self-management of type 2 diabetes: multicenter pragmatic randomized controlled trial. JMIR Mhealth Uhealth. 2019;7: e10321.

143. Lee JY, Kim JY, You SJ, Kim YS, Koo HY, Kim JH, et al. Development and usability of a life-logging behavior monitoring application for obese patients. J Obes Metab Syndr. 2019;28: 194.

144. Carter L, Rogith D, Franklin A, Myneni S. NewCope: a theory-linked mobile application for stress education and management. Stud Health Technol Inform. 2019;264: 1150.

145. Maramis C, Mylonopoulou V, Stibe A, Isomursu M, Chouvarda I. Developing a smartphone application to support smoking behavior change through social comparison. 2019 41st Annual International Conference of the IEEE Engineering in Medicine and Biology Society (EMBC). 2019. pp. 6922–6925.

146. Hébert ET, Ra CK, Alexander AC, Helt A, Moisiuc R, Kendzor DE, et al. A mobile just-in-time adaptive intervention for smoking cessation: pilot randomized controlled trial. J Med Internet Res. 2020;22: e16907. doi:10.2196/16907

147. Collins DAJ, Harvey SB, Lavender I, Glozier N, Christensen H, Deady M. A pilot evaluation of a smartphone application for workplace depression. Int J Environ Res Public Health. 2020;17: 6753. doi:10.3390/ijerph17186753

148. Torous J, Wisniewski H, Bird B, Carpenter E, David G, Elejalde E, et al. Creating a digital health smartphone app and digital phenotyping platform for mental health and diverse healthcare needs: an interdisciplinary and collaborative approach. J Technol Behav Sci. 2019;4: 73–85. doi:10.1007/s41347-019-00095-w

149. O’Grady C, Melia R, Bogue J, O’Sullivan M, Young K, Duggan J, et al. A mobile health approach for improving outcomes in suicide prevention (SafePlan). J Med Internet Res. 2020;22: e17481. doi:10.2196/17481

150. Purkayastha S, Addepally SA, Bucher S, others. Engagement and usability of a cognitive behavioral therapy mobile app compared with web-based cognitive behavioral therapy among college students: randomized heuristic trial. JMIR Hum Factors. 2020;7: e14146.

151. Okajima I, Akitomi J, Kajiyama I, Ishii M, Murakami H, Yamaguchi M. Effects of a tailored brief behavioral therapy application on insomnia severity and social disabilities among workers with insomnia in Japan: a randomized clinical trial. JAMA Netw Open. 2020;3: e202775–e202775. doi:10.1001/jamanetworkopen.2020.2775

152. Haug S, Castro RP, Wenger A, Schaub MP. Efficacy of a smartphone-based coaching program for addiction prevention among apprentices: study protocol of a cluster-randomised controlled trial. BMC Public Health. 2020;20: 1–8. doi:10.1186/s12889-020-09995-6

153. Nordholt PU, Christalle E, Zill JM, Dirmaier J. Engagement with a web-based intervention to reduce harmful drinking: secondary analysis of a randomized controlled trial. J Med Internet Res. 2020;22: e18826. doi:10.2196/18826

154. Litvin S, Saunders R, Maier MA, Lüttke S. Gamification as an approach to improve resilience and reduce attrition in mobile mental health interventions: a randomized controlled trial. PLoS One. 2020;15: e0237220. doi:10.1371/journal.pone.0237220

155. Casals M, Gangolells M, Macarulla M, Forcada N, Fuertes A, Jones R V. Assessing the effectiveness of gamification in reducing domestic energy consumption: Lessons learned from the EnerGAware project. Energy Build. 2020;210: 109753. doi:10.1016/j.enbuild.2019.109753

156. Rodante DE, Kaplan MI, Olivera Fedi R, Gagliesi P, Pascali A, José Quintero PS, et al. CALMA, a mobile health application, as an accessory to therapy for reduction of suicidal and non-suicidal self-injured behaviors: a pilot cluster randomized controlled trial. Archives of suicide research. 2020; 1–18. doi:10.1080/13811118.2020.1834476

157. Provoost S, Kleiboer A, Ornelas J, Bosse T, Ruwaard J, Rocha A, et al. Improving adherence to an online intervention for low mood with a virtual coach: study protocol of a pilot randomized controlled trial. Trials. 2020;21: 1–12. doi:10.1186/s13063-020-04777-2

158. Piao M, Ryu H, Lee H, Kim J, others. Use of the healthy lifestyle coaching chatbot app to promote stair-climbing habits among office workers: exploratory randomized controlled trial. JMIR Mhealth Uhealth. 2020;8: e15085. doi:10.2196/15085

159. Linardon J, Shatte A, Rosato J, Fuller-Tyszkiewicz M. Efficacy of a transdiagnostic cognitive-behavioral intervention for eating disorder psychopathology delivered through a smartphone app: a randomized controlled trial. Psychol Med. 2020; 1–12. doi:10.1017/S0033291720003426

160. De Marchi ACB, Alves ALS, Gonçalves CBC, Cervi CR, Biduski D, Bellei EA, et al. An electronic health platform for monitoring health conditions of patients with hypertension in the Brazilian public health system: protocol for a nonrandomized controlled trial. JMIR Res Protoc. 2020;9: e15299. doi:10.2196/15299

161. Simpson SA, Matthews L, Pugmire J, McConnachie A, McIntosh E, Coulman E, et al. An app-, web-and social support-based weight loss intervention for adults with obesity: the HelpMeDoIt! feasibility RCT. Public Health Research. 2020;8.

162. Klein A, Clucas J, Krishnakumar A, Ghosh SS, Van Auken W, Thonet B, et al. Remote digital psychiatry for mobile mental health assessment and therapy: MindLogger platform development study. J Med Internet Res. 2021;23: e22369. doi:10.2196/22369

163. Bendig E, Erb B, Meißner D, Bauereiß N, Baumeister H. Feasibility of a Software agent providing a brief Intervention for Self-help to Uplift psychological wellbeing (“SISU”). A single-group pretest-posttest trial investigating the potential of SISU to act as therapeutic agent. Internet Interv. 2021;24: 100377. doi:10.1016/j.invent.2021.100377

164. Eggink E, Hafdi M, Hoevenaar-Blom MP, Song M, Andrieu S, Barnes LE, et al. Prevention of dementia using mobile phone applications (PRODEMOS): protocol for an international randomised controlled trial. BMJ Open. 2021;11: e049762. doi:10.1136/bmjopen-2021-049762

165. Wei KS, Ibrahim NE, Kumar AA, Jena S, Chew V, Depa M, et al. Habits heart app for patient engagement in heart failure management: pilot feasibility randomized trial. JMIR Mhealth Uhealth. 2021;9: e19465. doi:10.2196/19465

166. Young CL, Mohebbi M, Staudacher HM, Kay-Lambkin F, Berk M, Jacka FN, et al. Optimizing engagement in an online dietary intervention for depression (My Food & Mood Version 3.0): Cohort study. JMIR Ment Health. 2021;8: e24871. doi:10.2196/24871

167. Ben-Zeev D, Chander A, Tauscher J, Buck B, Nepal S, Campbell A, et al. A smartphone intervention for people with serious mental illness: fully remote randomized controlled trial of CORE. J Med Internet Res. 2021;23: e29201.

168. Amanvermez Y, Karyotaki E, Cuijpers P, Salemink E, Spinhoven P, Struijs S, et al. Feasibility and acceptability of a guided internet-based stress management intervention for university students with high levels of stress: Protocol for an open trial. Internet Interv. 2021;24: 100369. doi:10.1016/j.invent.2021.100369

169. Raevuori A, Vahlberg T, Korhonen T, Hilgert O, Aittakumpu-Hyden R, Forman-Hoffman V. A therapist-guided smartphone app for major depression in young adults: a randomized clinical trial. J Affect Disord. 2021;286: 228–238. doi:10.1016/j.jad.2021.02.007

170. Coughlin LN, Nahum-Shani I, Philyaw-Kotov ML, Bonar EE, Rabbi M, Klasnja P, et al. Developing an adaptive mobile intervention to address risky substance use among adolescents and emerging adults: usability study. JMIR Mhealth Uhealth. 2021;9: e24424.

171. Hwang H, Bae S, Hong JS, Han DH, others. Comparing effectiveness between a mobile app program and traditional cognitive behavior therapy in obsessive-compulsive disorder: evaluation study. JMIR Ment Health. 2021;8: e23778. doi:10.2196/23778

172. Everitt N, Broadbent J, Richardson B, Smyth JM, Heron K, Teague S, et al. Exploring the features of an app-based just-in-time intervention for depression. J Affect Disord. 2021;291: 279–287. doi:10.1016/j.jad.2021.05.021

173. Osma López JJ, Mart\’\inez Garc\’\ia L, Peris Baquero Ó, Navarro Haro MV, González Pérez A, Suso-Ribera C. Implementation, efficacy and cost effectiveness of the unified protocol in a blended format for the transdiagnostic treatment of emotional disorders: a study protocol for a multicentre, randomised, superiority controlled trial in the Spanish National Health System. 2021. doi:10.1136/bmjopen-2021-054286

174. Rich RM, Ogden J, Morison L. A randomized controlled trial of an app-delivered mindfulness program among university employees: effects on stress and work-related outcomes. Int J Workplace Health Manag. 2021. doi:10.1108/IJWHM-04-2020-0046

175. Ho FK, Tung KTS, Wong RS, Chan KL, Wong WHS, Ho SY, et al. An Internet Quiz Game Intervention for Adolescent Alcohol Drinking: A Clustered RCT. Pediatrics. 2021;148. doi:10.1542/peds.2021-051005

176. Cerea S, Ghisi M, Bottesi G, Manoli T, Carraro E, Doron G. Cognitive behavioral training using a mobile application reduces body image-related symptoms in high-risk female university students: a randomized controlled study. Behav Ther. 2021;52: 170–182. doi:10.1016/j.beth.2020.04.002

177. Kraft R, Idrees AR, Stenzel L, Nguyen T, Reichert M, Pryss R, et al. eSano–An eHealth platform for internet-and mobile-based interventions. 2021 43rd Annual International Conference of the IEEE Engineering in Medicine & Biology Society (EMBC). 2021. pp. 1997–2002. doi:10.1109/EMBC46164.2021.9629534

178. Trottier CF, Lieffers JRL, Johnson ST, Mota JF, Gill RK, Prado CM. The impact of a web-based mindfulness, nutrition, and physical activity platform on the health status of first-year university students: protocol for a randomized controlled trial. JMIR Res Protoc. 2021;10: e24534. doi:10.2196/24534

179. Akin-Sari B, Inozu M, Haciomeroglu AB, Cekci BC, Uzumcu E, Doron G. Cognitive Training via a mobile application to reduce obsessive-compulsive-related distress and cognitions during the COVID-19 outbreaks: a randomized controlled trial using a subclinical cohort. Behav Ther. 2022. doi:10.1016/j.beth.2021.12.008

180. Cobos-Campos R, Apiñaniz A, de Lafuente AS, Parraza N. Development, validation and transfer to clinical practice of a mobile application for the treatment of smoking. Aten Primaria. 2022;54: 102363. doi:10.1016/j.aprim.2022.102363

181. Mueller J, Richards R, Jones RA, Whittle F, Woolston J, Stubbings M, et al. Supporting Weight Management during COVID-19: A Randomized Controlled Trial of a Web-Based, ACT-Based, Guided Self-Help Intervention. Obes Facts. 2022; 1–10. doi:10.1159/000524031

182. Graham AL, Papandonatos GD, Cha S, Amato MS, Jacobs MA, Cohn AM, et al. Effectiveness of an optimized text message and Internet intervention for smoking cessation: A randomized controlled trial. Addiction. 2022;117: 1035–1046. doi:10.1111/add.15677

183. Varga A, Czeglédi E, Tóth MD, Purebl G. Effectiveness of iFightDepression® online guided self-help tool in depression–A pilot study. J Telemed Telecare. 2022; 1357633X221084584. doi:10.1177/1357633X221084584

184. Hoepper BB, Siegel KR, Carlon HA, Kahler CW, Park ER, Taylor ST, et al. Feature-Level Analysis of a Smoking Cessation Smartphone App Based on a Positive Psychology Approach: Prospective Observational Study. JMIR Form Res. 2022;6: e38234. doi:10.2196/38234

185. McGillivray L, Gan DZQ, Wong Q, Han J, Hetrick S, Christensen H, et al. Three-arm randomised controlled trial of an m-health app and digital engagement strategy for improving treatment adherence and reducing suicidal ideation in young people: study protocol. BMJ Open. 2022;12: e058584.

186. Goulding EH, Dopke CA, Rossom RC, Michaels T, Martin CR, Ryan C, et al. A Smartphone-Based Self-management Intervention for Individuals With Bipolar Disorder (LiveWell): Empirical and Theoretical Framework, Intervention Design, and Study Protocol for a Randomized Controlled Trial. JMIR Res Protoc. 2022;11: e30710. doi:10.2196/30710

187. Saur S, Weisel KK, Lang C, Fuhrmann LM, Steins-Loeber S, Enewoldsen N, et al. App-based maintenance treatment for alcohol use disorder after acute inpatient treatment: Study protocol for a multicentre randomized controlled trial. Internet Interv. 2022;28: 100517. doi:10.1016/j.invent.2022.100517

188. Chaves A, Arnáez S, Castilla D, Roncero M, Garc\’\ia-Soriano G. Enhancing mental health literacy in obsessive-compulsive disorder and reducing stigma via smartphone: A randomized controlled trial protocol. Internet Interv. 2022; 100560. doi:10.1016/j.invent.2022.100560

189. Wang X, Wang S, Zhang C, Zhong L, Lerman L, Lerman A, et al. Protocol: Internet-based platform for a low-calorie dietary intervention involving prepackaged food for weight loss in overweight and obese individuals in China: protocol for a randomised controlled trial. BMJ Open. 2022;12.

190. Thabrew H, Boggiss AL, Lim D, Schache K, Morunga E, Cao N, et al. Well-being app to support young people during the COVID-19 pandemic: randomised controlled trial. BMJ Open. 2022;12: e058144. doi:10.1136/bmjopen-2021-058144

191. Dietvorst E, Aukes MA, Legerstee JS, Vreeker A, Hrehovcsik MM, Keijsers L, et al. A Smartphone Serious Game for Adolescents (Grow It! App): Development, Feasibility, and Acceptance Study. JMIR Form Res. 2022;6: e29832. doi:10.2196/29832

192. Agher D, Sedki K, Despres S, Albinet J-P, Jaulent M-C, Tsopra R. Encouraging behavior changes and preventing cardiovascular diseases using the prevent connect mobile health app: conception and evaluation of app quality. J Med Internet Res. 2022;24: e25384.
