# Supplementary material for: Persuasive technologies design for mental and behavioral health platforms: A scoping literature review
Source: PLOS Digit Health. 2024 May 16;3(5):e0000498. doi: 10.1371/journal.pdig.0000498 (PMC11098517; doi:10.1371/journal.pdig.0000498)
Supplement: S2 Table — (DOCX) [file pdig.0000498.s002.docx]

**S2 Table.** Synthesis for the PD frameworks

| **Framework** | **User understanding** | **System interaction** | **Persuasion techniques** | **Social and trust factors** |
| --- | --- | --- | --- | --- |
| FBM [10] | Behavioral components | Behavior components | Motivation, ability, triggers | - |
| PSD [13] | User-centric feature selection | Primary task, dialogue, credibility features | List of persuasive strategies | Credibility and social support features |
| Fogg’s eight-step [15] | Implicit user understanding in design | Step-by-step interaction guide | Outcome to experience design | Implicit trust in design |
| Kimura and Nakajima [16] | Understands collectivist cultural issues | Thematic focus on collectivist persuasion | Collectivist cultural persuasion | Emphasis on social context |
| 3D-RAB [17] | user psychographics | Tailored system features | Tailored persuasion techniques | Trust and social dynamics consideration |
| CPP [18] | Tailors strategies to user base and context | Strategy tailoring to context | Strategy selection and tailoring | Tailoring influences trust |
| U-FADE [19] | Determines factors influencing behavior | Selection of System Features from PSD model. | Incorporates persuasive strategies from PSD model | Credibility and social support features |
| Al-Ramahi [20] | - | Self-monitoring and informative presentation | effort expectancy and persuasive messages | Emphasizes peer community support |
| PEM [21] | Recognizes psychological state of users across stages | Mapping system functions to psychological stages | Stages represent different persuasion levels | - |
| Schneide et al. [22] | Identifies user categories (Followers, Hedonists, Achievers) | Tailored interactions for different user categories | Perceived Behavioral Control; Support Flexibility | Followers benefit from mutual encouragement |
| Murillo-Munoz et al. [23] | Understanding key issues, contextual inquiry, affinity diagramming, personas | system qualities design, mockup evaluation, software cycle, software release | Analyzing persuasion context, system qualities design from PSD model | Participatory design phase |
| Alpay et al. [24] | Identifying stakeholders, shaping requirements | Strategically addressing user needs | Leverages PSD model and Fogg Behavior Model | - |
| Sari et al. [25] | Define the behavioral issue and target behavior | - | Behavior Change Wheel (BCW) for target behavior; integrate PSD model strategies | Credibility and social support features |
| EMVE-DeCK [26] | Understand the target behavior | System design, deployment, and documentation. | Grounded in the PSD model. | Credibility and social support features |
| Demonte and Souto [27] | Context Identification; Interface Design | Suitability to Context | Persuasion strategies; draws from PSD model and FBM | Credibility and social support features |
