# Supplementary material for: Persuasive technologies design for mental and behavioral health platforms: A scoping literature review
Source: PLOS Digit Health. 2024 May 16;3(5):e0000498. doi: 10.1371/journal.pdig.0000498 (PMC11098517; doi:10.1371/journal.pdig.0000498)
Supplement: S3 Table — (DOCX) [file pdig.0000498.s003.docx]

**S3 Table.** Studies evaluation

| **Article** | **Framework** | **Outcome** | **Bias** | | |
| --- | --- | --- | --- | --- | --- |
|  |  |  | **Selection** | **Reporting** | **Detection** |
| Kimura and Nakajima [16] | Kimura and Nakajima | Mixed | H^*^ | M^*^ | M |
| Sankaran et al. [30] | PSD model/FBM | Mixed | H | M | M |
| Karim et al. [31] | PSD model/FBM | Positive | H | M | M |
| Mohadis et al. [32] | PSD model | Mixed | H | M | M |
| Woldemicael [35] | PSD model | Mixed | H | M | M |
| Tikka et al. [36] | PSD model | Positive | M | M | M |
| Akker et al. [38] | PSD model | Positive | M | M | M |
| Ladwa et al. [41] | PSD model | Positive | H | M | M |
| Häkkä [43] | PSD model | Mixed | H | M | M |
| Renfrew et al. [44] | PSD model | Mixed | L^*^ | L | M |
| Oyebode et al. [45] | PSD model | Positive | L | L | L |
| Merz and Hurm [47] | PSD model | Positive | H | M | H |
| Fenicio and Calvary [48] | FBM | Mixed | H | M | M |
| Akmal and Niwanputri [49] | FBM | Positive | L | L | L |
