# Supplementary material for: Persuasive technologies design for mental and behavioral health platforms: A scoping literature review
Source: PLOS Digit Health. 2024 May 16;3(5):e0000498. doi: 10.1371/journal.pdig.0000498 (PMC11098517; doi:10.1371/journal.pdig.0000498)
Supplement: S1 Text — (DOCX) [file pdig.0000498.s004.docx]

# S1 Text: Detailed Search Strings

This provides the detailed search strings used for the systematic review to ensure reproducibility.

## Databases Searched

The following databases were included in our search:

- PubMed
- IEEE Xplore digital library
- Journal of Medical Internet Research
- Google-Scholar
- ScienceDirect
- ACM Digital Library
- The Web of Science
- Oxford University Press

## Search Strings

The main search strings used across the databases are:

- ‘persuasive design framework$’
- ‘persuasive design strateg*’
- ‘persuasive design architecture$’
- ‘persuasive design methodolog*’
- ‘eHealth platform$’
- ‘eHealth app*’
- ‘eHealth intervention$’
- ‘internet intervention$’
- ‘internet-based intervention$’
- ‘internet and mobile-based intervention$’
- ‘digital health intervention$’
- ‘computerized CBT’
- ‘persuasive design’
- ‘persuasive technolog*’
- ‘behaviour change’
- ‘behavior change’
- ‘gamification’
